# Autologous Platelet- and Extracellular Vesicle-Rich Plasma Is an Effective Treatment Modality for Chronic Postoperative Temporal Bone Cavity Inflammation: Randomized Controlled Clinical Trial

**DOI:** 10.3389/fbioe.2021.677541

**Published:** 2021-07-07

**Authors:** Domen Vozel, Darja Božič, Marko Jeran, Zala Jan, Manca Pajnič, Ljubiša Pađen, Nejc Steiner, Veronika Kralj-Iglič, Saba Battelino

**Affiliations:** ^1^Department of Otorhinolaryngology and Cervicofacial Surgery, University Medical Centre Ljubljana, Ljubljana, Slovenia; ^2^Faculty of Medicine, University of Ljubljana, Ljubljana, Slovenia; ^3^Laboratory of Clinical Biophysics, Faculty of Health Sciences, University of Ljubljana, Ljubljana, Slovenia; ^4^Laboratory of Physics, Faculty of Electrical Engineering, University of Ljubljana, Ljubljana, Slovenia; ^5^Extracellular Vesicles and Mass Spectrometry Laboratory, Institute of Biosciences and BioResources, National Research Council of Italy, Naples, Italy

**Keywords:** regenerative medicine, mastoidectomy, cholesteatoma, theranostic nanomedicine, quality of life, temporal bone, platelet-rich plasma, extracellular vesicles

## Abstract

**Purpose:**

To determine the efficacy of autologous platelet- and extracellular vesicle-rich plasma (PVRP) to treat chronic postoperative temporal bone cavity inflammation (CPTBCI) after exhausting surgical and standard conservative therapies.

**Materials and Methods:**

Patients were randomly allocated to treatment with PVRP (PVRP group) or standard conservative methods (control group) in a setting of four once-monthly checkups and subsequent follow-up. The treatment outcome was measured with the Chronic Otitis Media Questionnaire-12 (COMQ-12), CPTBCI focus surface area, and CPTBCI symptom-free time after the fourth checkup.

**Results:**

Eleven patients from each group completed the trial; 95% of patients suffered from chronically discharging mastoid cavity (the type of CPTBCI). Within four checkups, the COMQ-12 score decreased statistically significantly in the PVRP group (*p* < 0.001) but not in the control group (*p* = 0.339). The CPTBCI foci surface area decreased statistically significantly between the first and second checkups (*p* < 0.0005) but not between other checkups (*p* > 0.05) in the PVRP group. No statistically significant differences in CPTBCI foci surface area were detected between checkups in the control group (*p* = 0.152). Nine patients from the PVRP group and three patients from the control group were CPTBCI symptom-free at the fourth checkup. The median symptom-free time was 9.2 months (95% CI [7.4, 11.9]) in the PVRP group. Cumulatively, 49% of patients in the PVRP group remained CPTBCI symptom-free for 12.7 months after the fourth checkup.

**Conclusion:**

Autologous PVRP represents a novel additional and successful treatment modality for a chronically discharging radical mastoid cavity when the surgical and standard conservative treatment methods have been exhausted.

**Trial Number:**

https://clinicaltrials.gov (NCT04281901).

## Introduction

The chronic postoperative temporal bone cavity inflammation (CPTBCI) typically implies a chronically discharging radical mastoid cavity, which results from canal wall down (i.e., radical) mastoidectomy (Gluth et al., 2013). This otological surgical procedure is performed in about 53% of cholesteatoma surgery (Wilkie et al., 2019). Although the CPTBCI affects only 1.5% of patients with the radical mastoid cavity (Pareschi et al., 2019), its burden on quality of life and healthcare is significant (Khalil and Windle-Taylor, 2003; Dornhoffer et al., 2008). The treatment is challenging, especially when the established treatment methods are exhausted. Considering that the CPTBCI is a subtype of chronic otitis media (COM), health-related quality of life can be assessed with patient-reported outcome measures, such as the Chronic Otitis Media Questionnaire-12 (COMQ-12) (Phillips et al., 2014; Demir et al., 2020).

The awareness of the burden of CPTBCI has induced research in novel treatment modalities, including the application of medical honey and the refinement of surgical techniques (Henatsch et al., 2015). On the other hand, the platelet-rich plasma (PRP) has been widely used to promote wound healing in many clinical settings, recently in skull-base reconstruction (Khafagy et al., 2018), nasal septal surgery, tonsillectomy (Gökçe Kütük and Özdaş, 2019), and laryngectomy (Eid et al., 2020) due to beneficial immune, hemostatic, and regenerative effects of platelets exerted by the release of growth factors from granules and several extra-granular molecules. Despite that, research, such as PRP in otorhinolaryngology, lacks randomized controlled clinical trials (Pachito et al., 2019; Vozel et al., 2020b). As plasma contains extracellular vesicles (EVs), which are considered to be important healing vectors, we refer to the preparation as platelet- and extracellular vesicle-rich plasma (PVRP) in this study (Tao et al., 2017). EVs are a heterogeneous group of nanosized cell-derived membrane vesicles with essential roles in intercellular communication and possess an exceptional potential to be used in diagnosis, treatment, or both (i.e., theranostics) (Yáñez-Mó et al., 2015). EVs are naturally present in the blood. In addition, they are produced from cells in blood during the sampling and processing of samples (Šuštar et al., 2011). Although the concentrations of EVs in PVRP are expected to be high, the literature describing this is scarce (Guo et al., 2017).

For the abovementioned reasons, the importance of research on the efficacy of PVRP in the treatment of CPTBCI has been recognized. As a result, this prospective randomized controlled clinical trial aims to compare the efficacy of autologous PVRP (administered via ear wick) and standard conservative methods in the CPTBCI treatment by measuring the outcome at 1, 2, and 3 months after the baseline evaluation with the CPTBCI surface area measurement and COMQ-12. The trial hypothesizes that the PVRP is efficient in the CPTBCI treatment when standard conservative and surgical methods have been exhausted. The exhaustion of methods to treat CPTBCI is the rationale behind this trial.

## Materials and Methods

The trial was registered, and results were reported on clinicaltrials.gov (No. NCT04281901) and approved by the National Medical Ethics Committee Slovenia (No. 0120-146/2019/5). Written informed consent was obtained from all individual participants included in the study.

### Enrolment

**FIGURE 1:**
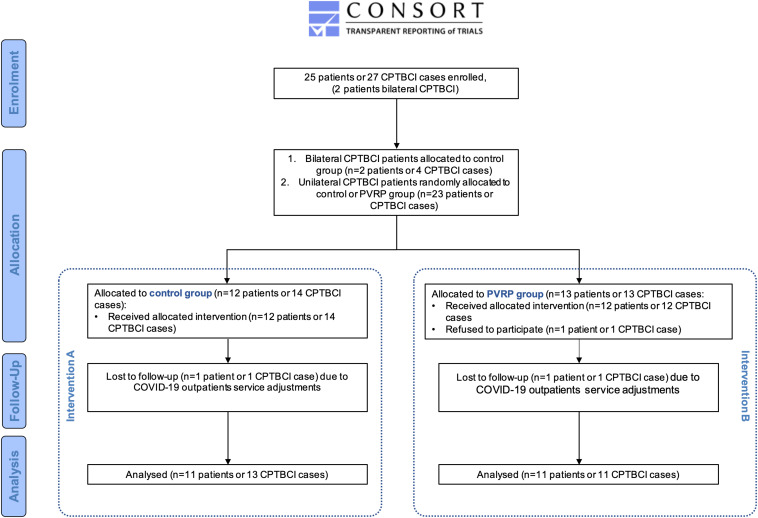
Flowchart of a prospective randomized controlled clinical trial for treating patients with chronic postoperative temporal bone cavity inflammation. Data are presented according to the CONSORT statement (Schulz et al., 2010). Control group represented intervention A and PVRP intervention B. CPTBCI: chronic postoperative temporal bone cavity inflammation; PVRP: platelet- and extracellular vesicle-rich plasma; COVID-19: coronavirus disease 2019.

The trial started by patient enrolment (Figure 1) during regularly scheduled checkups between March 20, 2019 and September 15, 2019 at the tertiary otorhinolaryngology referral center by leading researchers (SB and DV). This time is considerably longer than the period between two checkups in a patient with CPTBCI.

The inclusion criteria of the trial were:

• Age > 18 years.
• COM defined as a presence of ≥ 1 of clinical signs of inflammation exacerbation, visible ear discharge, indirect ear discharge signs (e.g., on a pillow and clothes), itching, and the sensation of ear fullness.
• Exhausted surgical treatment: additional surgical treatment of CPTBCI would not preserve serviceable hearing loss.
• Exhausted standard conservative treatment: ineffective conservative treatment of CPTBCI for at least 8 weeks, which included administration of antimicrobial, anti-inflammatory, antiseptic preparations (i.e., drops, ointments, pastes, and powders), and/or silver nitrate sticks (to perform chemocautery on the granulation tissue).

The exclusion criteria of the trial were:

• Venipuncture site inflammation.
• Pregnancy or breastfeeding, chronic use of immunomodulatory, and/or antimicrobial drugs.
• Presence of systemic infectious disease, autoimmune disease, and malignancy.
• Other experimental attempts to treat CPTBCI.
• Inability and/or refusal of the patient to participate in the trial.

Unilateral CPTBCI was considered as one case and bilateral CPTBCI as two cases of CPTBCI.

### Allocation and Treatment Initiation

After enrolment, patients were allocated treatment with standard conservative methods (i.e., control group) or treatment with PVRP (i.e., PRVP group) with simple random allocation (Machin and Fayers, 2010) using sealed envelopes by a senior researcher (VK-I) (Supplementary Material 1). Then, patients were informed by the leading researchers (DV and SB) about the treatment allocation at their next checkup scheduled regularly between September 18, 2019 and January 22, 2020. After re-evaluating the inclusion and exclusion criteria of the trial at this checkup, informed written consent was provided, and the patient started to participate in the trial. Later, the treatment regimen of the trial, which included four checkups, was initiated. After the first (i.e., baseline) checkup, three checkups with 4-week intervals were planned by the leading researchers (DV and SB). Subsequent checkups (i.e., after the fourth checkup) were considered as a follow-up.

The control group was treated with standard conservative methods (described in the inclusion criteria of the trial) at each checkup. The PVRP group was treated with the autologous PVRP at the first and second checkups. The aural toilet (i.e., cleaning by aspiration) was performed in all patients at each checkup. All patients were instructed to comply with the dry ear precautions.

Refusal of a patient to participate in the trial did not change the regular management of CPTBCI.

### Primary Outcome Measures

At each of the four checkups, the CPTBCI focus surface area was measured for each CPTBCI case, and every patient completed the COMQ-12 (Phillips et al., 2014). The latter was validated and cross-culturally adapted in the target language (Vozel et al., 2020c).

**FIGURE 2:**
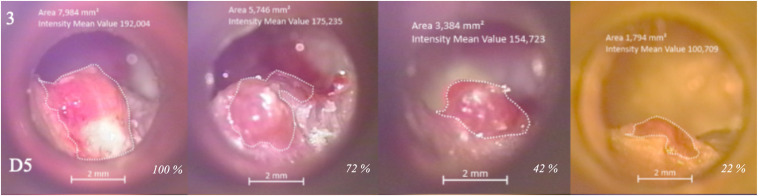
Chronic postoperative temporal bone cavity inflammation focus surface area measurements. Otomicroscopical photographs of CPTBCI (i.e., chronically discharging radical mastoid cavity) in a patient at four checkups depict surface areas (mm^2^) transformed to percentages (italics) according to the baseline. The figure represents a regression of inflammation (i.e., spherical granulation) in a patient treated with PVRP. D5 marks the anatomical area of CPTBCI focus according to the anatomical classification (Supplementary Material 2) and in the upper left corner, an ID of the patient (Supplementary Materials 7–10). CPTBCI: chronic postoperative temporal bone cavity inflammation; PVRP: platelet- and extracellular vesicle-rich plasma.

The CPTBCI foci (e.g., granulation tissue, redness, and edema) were detected and photographed by the leading researchers (DV and SB) with a diagnostic otomicroscope (OPMI pico/S100, Carl Zeiss Surgical GmbH, Oberkochen, Germany) at 5.1× and 8.5× magnification, for which calibration was performed. The focus of inflammation was instantly anatomically classified (Supplementary Material 2). Later, the independent blinded outcome assessor (NS), unaware of treatment allocation, outlined the focus and measured the CPTBCI focus surface using the ZEN 3.0 blue edition software (© Carl Zeiss Microscopy GmbH, 2019). Areas in mm^2^ were converted into a percentage with respect to the baseline CPTBCI surface area (i.e., 100% at the first checkup) as depicted in Figure 2.

### Secondary Outcome Measure

The secondary outcome measure was CPTBCI symptom-free time measured in the follow-up period. It was analyzed in patients who did not suffer from CPTBCI-related symptoms between the fourth checkup and subsequent checkup(s) in the follow-up period. These symptoms were ear discharge, indirect ear discharge signs (e.g., on a pillow and clothes), itching, pain, and the sensation of ear fullness.

### Platelet- and Extracellular Vesicle-Rich Plasma-Related Adverse Events

The PVRP-related adverse events were considered as aggravation of CPTBCI when intensification of ear discharge, an itch, and a sensation of ear fullness occurred within 3 days after PVRP administration. To monitor the adverse events, patients were instructed to contact the leading researchers (DV and SB) or tertiary otorhinolaryngology referral center.

### Platelet- and Extracellular Vesicle-Rich Plasma Preparation Protocol

**FIGURE 3:**
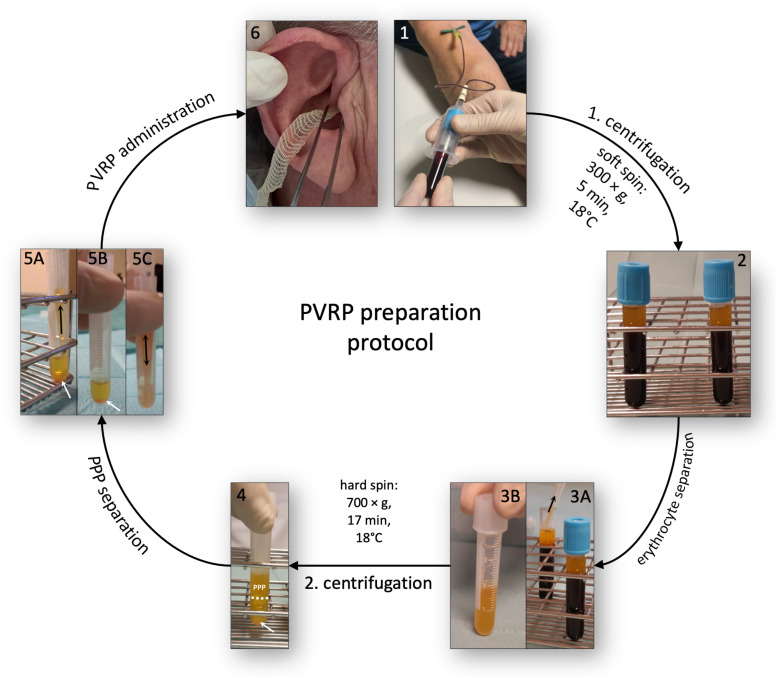
Autologous PVRP preparation protocol. Four citrated blood tubes were used to prepare two units of PVRP in parallel. Panels 1–6 denote preparation steps. PVRP: platelet- and extracellular vesicle-rich plasma; PPP: platelet-poor plasma. Adapted from Vozel et al. (2020b).

Blood was drawn into four 4.5-ml evacuated blood tubes with sodium citrate anticoagulant (9 NC sodium citrate 0.105 M, BD Vacutainer, Becton Dickinson, San Jose, CA, United States, stored at room temperature) using a 21-G wing needle (Safety-Lok Blood Collection Set, BD Vacutainer, Becton Dickinson, San Jose, CA, United States) (Figure 3, panel 1). Two filled blood tubes were used to prepare one unit of PVRP, and two units were prepared in parallel. Blood tubes were immediately transported at ambient temperature (22–24°C) to the laboratory, where the PVRP preparation started. First, the soft spin centrifugation (i.e., 5 min, 300 × *g*, 18°C) of blood tubes was performed, and two fractions were distinguished (Figure 3, panels 2, 3A): hematocrit (red layer in the bottom) and plasma with platelets (yellow upper layer). Occasionally, a buffy coat (thin opaque whitish layer of leukocytes) on top of hematocrit could be observed. Plasma directly above the buffy coat was transferred by a sterile pipette (Figure 3, panel 3A) into a sterile polypropylene tube (Figure 3, panel 3B). Caution was required to prevent mixing layers, i.e., disruption of hematocrit and buffy coat and consequent leukocyte and erythrocyte harvesting. Collected plasma was equally distributed among two sterile polypropylene tubes and then centrifuged at hard spin (i.e., 17 min, 700 × *g*, 18°C) to sediment the platelets plasma (Figure 3, white arrows in panels 4, 5A, and 5B). The upper half of the centrifuged plasma (bordered by the white dashed line Figure 3, panel 4), called platelet-poor plasma, was carefully removed and discarded without disruption of layers (Figure 3, panel 5A). The remaining pellet was resuspended in the remaining plasma volume (Figure 3, panel 5B) in the same tube (Figure 3, panel 5C) to produce PVRP. Two units of PVRP were pooled and administered to the same patient (i.e., autologously) via ear wick (i.e., gauze tamponade 1 cm × 10 m cut on a 7-cm long wick, Tosama d.o.o., Domzale, Slovenia) into the area of CPTBCI (Figure 3, panel 6). The patient was instructed to remove the ear wick after 2 days. Autologous PVRP administration was performed at the first and second checkups in the PVRP group.

### Analyses of Blood and Platelet- and Extracellular Vesicle-Rich Plasma

Concentration of leukocytes, erythrocytes, hemoglobin, C-reactive protein (CRP), differential blood cell count, and erythrocyte sedimentation rate (ESR) was determined in blood samples by standard automatic cytometry methods.

Additionally, blood and PVRP samples were analyzed by flow cytometry (flow cytometer MACS QUANT, Miltenyi, Bergisch-Gladbach, Germany, and the related MACSQuantify software) to classify PVRP as given in DeLong et al. (2012) according to the platelet concentrations in PVRP (DeLong et al., 2012). Particles were characterized based on forwarding and side scattering signals of the flow cytometer. The gates were set in a preliminary study, in which the identity of cells in the presented regions was confirmed by immuno-labeling and by scanning electron microscopy analysis. The latter also provided evidence on the presence of vesicles. By flow cytometry approaches used in this study, a fraction of vesicles (bigger than the threshold 400 nm) is detected and evaluated as a part of the population P2 (Supplementary Material 3). Later, the yield of platelets and the detectable fraction of EVs in PVRP was calculated by the following equation:

(1) $$\frac{\mathrm{concentration}\,\mathrm{of}\,\mathrm{platelets}\,\mathrm{and}\,\mathrm{EVs}\,\mathrm{in}\,\mathrm{PVRP}}{\mathrm{concentration}\,\mathrm{of}\,\mathrm{platelets}\,\mathrm{and}\,\mathrm{EVs}\,\mathrm{in}\,\mathrm{blood}}\;$$

The PVRP was prepared for sterility analysis using the preparation protocol described above from volunteers aged over 18 years and without CPTBCI and associated diseases and signs of infection.

Chemicals, additional materials, and methods used in the PVRP analysis are described in Supplementary Material 4.

### Statistical Analysis

A Microsoft Excel for Mac (versions 16.9.0-16.36, Microsoft Corp., Redmond, WA, United States) and IBM SPSS (version 23, IBM Corp., Armonk, NY, United States) was used for statistical analysis. The *p*-value < 0.05 was considered statistically significant. Additional description of statistical analysis is provided in Supplementary Material 5.

## Results

Results of the interim analyses of the trial have been partially presented elsewhere (Vozel et al., 2020a).

### Patients

The trial flowchart is depicted in Figure 1 and reported according to the CONSORT statement in Supplementary Material 6 (Schulz et al., 2010). Twenty-five patients or 27 cases of CPTBCI (two patients with bilateral involvement) were enrolled and allocated. One patient allocated to the PVRP group refused to participate in the trial, and two (i.e., one from each group) were lost to follow-up. The final analysis included 22 patients or 24 cases of CPTBCI (i.e., two patients with bilateral CPTBCI) (Table 1 and Supplementary Material 7).

**TABLE 1:** Baseline characteristics of the patient.

	PVRP group (*n* = 11)	Control group (*n* = 11)	*p*
**Gender**			
Male (*n*, %)	9 (82%)	9 (82%)	1.000
Female (*n*, %)	2 (18%)	2 (18%)	
**Age**			
*M* ± SD (years)	47 ± 18	52 ± 24	0.632^‡^
**Comorbidities**			
Yes (*n*, %)	6 (55%)	6 (55%)	1.000
No (*n*, %)	5 (45%)	5 (45%)	
**Regular medicine intake**			
Yes (*n*, %)	3 (27%)	6 (55%)	0.387*
No (*n*, %)	8 (73%)	5 (45%)	
**Allergies**			
Yes (*n*, %)	5 (45%)	4 (36%)	1.000*
No (*n*, %)	6 (55%)	7 (64%)	
**Coagulopathy**			
Yes (*n*, %)	2 (18%)	1 (9%)	1.000*
No (*n*, %)	9 (82%)	10 (91%)	
**Smoking**			
Yes (*n*, %)	2 (18%)	5 (45%)	0.361*
No (*n*, %)	9 (82%)	6 (55%)	
**Alcohol consumption**			
Yes (*n*, %)	10 (91%)	7 (64%)	0.311*
No (*n*, %)	1 (9%)	4 (36%)	
**Time to treatment**			
Mdn (day–years)	2,688–7.4	3,172–8.7	0.116**
**Time from surgery**			
Mdn (days–years)	4,644–12.7	5,224–14.3	0.573**
**COMQ-12 score**			
Mdn	33	34	0.977**

Time to treatment – the time from the beginning of the problems due to chronic postoperative temporal bone cavity inflammation to the first checkup of the trial; time from surgery – the time from the last surgery required to treat chronic ear disease to the first checkup of the trial. PVRP: platelet- and extracellular vesicle-rich plasma; *n*: number of patients; *M*: average value; ^‡^independent samples *t*-test; *Fisher’s exact test; Mdn: median value; **Mann–Whitney *U*-test; COMQ-12: chronic otitis media questionnaire 12; *p*-value < 0.05 denotes statistically significant difference. Coagulopathy occurred as a consequence of regular acetylsalicylic acid intake.

In 21 patients (95%), i.e., 23 cases (96%), CPTBCI occurred as a chronically discharging radical mastoid cavity. In one patient (5%), i.e., one case (4%), CPTBCI occurred in a cavity after subtotal petrosectomy which was performed to treat the external auditory canal squamous cell carcinoma, which did not recur since 2004. One patient (5%), i.e., one case of CPTBCI (4%) in the PVRP group, underwent radical mastoidectomy due to external auditory canal cholesteatoma. In 20 patients (91%), i.e., 22 cases (92%) (10 cases from the PVRP group and 12 cases from the control group), radical mastoidectomy was performed due to middle ear cholesteatoma (Supplementary Material 7).

The intervals between checkups did not show statistically significant difference between groups (*Mdn* = 28 and *p* > 0.05 for each interval between checkups) (Supplementary Material 9).

Blood laboratory results determined by standard automatic cytometry methods were normal in all patients. The average platelet and EV concentration measured by flow cytometry in the blood samples of the PVRP group was 199 × 10^9^/L (SD = 62 × 10^9^/L) (Supplementary Material 8).

### Treatment Outcome

#### Chronic Otitis Media Questionnaire-12

**FIGURE 4:**
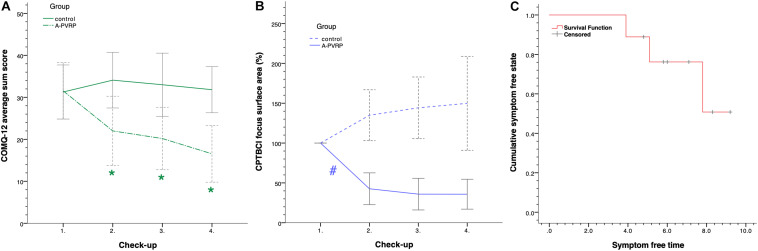
Treatment outcome and symptoms at follow-up. **(A)** COMQ-12 average sum scores (Vozel et al., 2020a); **(B)** CPTBCI focus area percentages; **(C)** Kaplan–Meier curve. 95% CIs are included in graphs **(A,B)**. Graph **(C)** is based on the data of 9 of 11 (82%) patients treated with PVRP who were symptom-free after the fourth checkup of the trial. Cumulatively, 49% of patients from the PVRP group remained symptom-free for 12.7 months after the fourth checkup. COMQ-12: chronic otitis media questionnaire 12; CPTBCI: chronic postoperative temporal bone cavity inflammation; PVRP: platelet- and extracellular vesicle-rich plasma; *statistically significant difference between groups within checkup; #statistically significant difference between the first and second checkups in the PVRP group.

Intervention allocation had a statistically significant effect on the COMQ-12 sum scores throughout four checkups according to the two-way mixed ANOVA [*F*(3, 60) = 8.755, *p* < 0.0005, partial η^2^ = 0.304] (Figure 4A, Supplementary Material 9). At the first checkup, there was no statistically significant difference in scores between groups [*F*(1, 20) = 0.00, *p* = 0.949], but the difference was statistically significant at the second [*F*(1, 20) = 6.48, *p* = 0.019, partial η^2^ = 0.245], third [*F*(1, 20) = 7.30, *p* = 0.014, partial η^2^ = 0.267] and fourth checkup [*F*(1, 20) = 15.29, *p* = 0.001, partial η^2^ = 0.433]. The score decreased statistically significantly during the treatment within the PVRP [*F*(3, 30) = 9.78, *p* < 0.001, partial η^2^ = 0.494] and not in the control group [*F*(3, 30) = 1.17, *p* = 0.339]. The decrease of the score was statistically significant in the PVRP group between the first and third (*M* = 11 points, SE = 3 points, *p* = 0.029) and between the first and fourth checkup (*M* = 15 points, SE = 2 points, *p* = 0.001), but not between other checkups (*p* > 0.05).

#### The CPTBCI Foci Surface Areas

Differences of CPTBCI foci surface areas within the PVRP group between the first (*M* = 100%, SD = 0%), second (*M* = 43%, SD = 30%), third (*M* = 36%, SD = 30%), and fourth checkups (*M* = 36%, SD = 28%) were statistically significant according to the one-way ANOVA with repeated measures [*F*(3, 30) = 28.59, *p* < 0.0005, partial *ω*^2^ = 0.650] (Figure 4B and Supplementary Materials 9, 10). According to the *post-hoc* analysis with the Bonferroni correction, the decrease was statistically significant between the first and second checkups (*M* = 57%, 95% CI [28, 87], *p* < 0.0005) but not between other check-ups (*p* > 0.05). No statistically significant differences were detected between checkups in the control group according to the one-way ANOVA with repeated measures corrected for violation of sphericity [correction with ε = 0.611, *F*(1.834, 22.010) = 2.079, *p* = 0.152].

#### Follow-up

At the fourth checkup, 82% (i.e., nine patients) from the PVRP group and 27% (i.e., three patients) from the control group were CPTBCI symptom-free; therefore, the CPTBCI symptom-free time was measured for these patients (Supplementary Material 9). Data were cut off on February 22, 2021 for the patients who were symptom-free at that date. The median symptom-free time was 9.2 months (95% CI [7.4, 11.9]) in the PVRP group. According to the Kaplan–Meier analysis, cumulatively, 49% of patients in the PVRP group remained symptom-free for 12.7 months after the fourth checkup (Figure 4C). About 89% of these patients remained symptom-free until 3.9 months, 78% until 5.1 months, and 65% until 7.8 months.

### Platelet- and Extracellular Vesicle-Rich Plasma-Related Adverse Events

No PVRP-related adverse events were detected.

### Platelet- and Extracellular Vesicle-Rich Plasma Preparation

The PVRP preparation, on average, took 80 min (SD = 26 min). The average volume of PVRP for one administration (i.e., two units of PVRP) was 2.1 ml (SD = 0.7 ml), enough to soak the ear wick each time entirely. The average concentration of platelets and EVs in PVRP was 504 × 10^9^/L (SD = 190 × 10^9^/L). The average yield of platelets and EVs in PVRP (Equation 1) was 276% (SD = 157%) (Supplementary Material 8).

**FIGURE 5:**
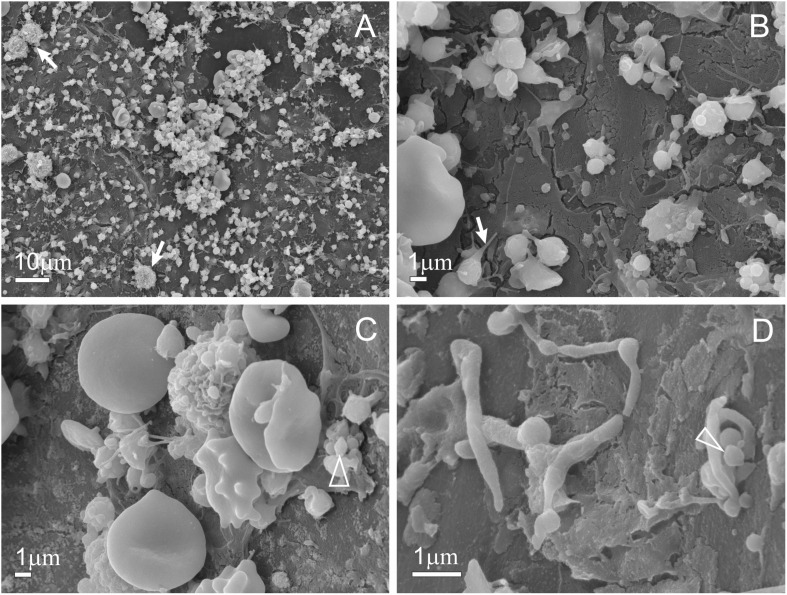
Scanning electron microscopy of PVRP. Panel **(A)** depicts high platelet and extracellular vesicle (EV) count. The white arrow on panel **(A)** depicts leukocytes, the white arrow on panel **(B)** depicts a platelet forming a tubular protrusion prior to EV shedding, triangles on panels **(C,D)** depict EVs. Panel **(D)** depicts platelets and EVs, which are globular fragments sized 300–1,000 nm, formed after platelet activation and shedding of their tubular protrusions. PVRP: platelet- and extracellular vesicle-rich plasma.

Scanning electron microscopy of PVRP samples showed an abundance of platelets, as demonstrated in Figure 5A. We detected no resting disk-like platelets in PVRP. Instead, observed platelets came in different sizes and shapes and exhibited tubular protrusions. Fragmented or deformed platelets that have lost a considerable part of their membrane are considered EVs (Figures 5C,D) (Tao et al., 2017) since there is no sharp boundary between platelets and EVs.

The PVRP preparation protocol sterility analyses of the PVRP prepared from eight volunteers showed the absence of bacterial or fungal growth in all eight samples.

## Discussion

This randomized controlled clinical trial compares the efficacy of autologous sterile PVRP and standard conservative methods in the CPTBCI treatment. Autologous PVRP possesses beneficial effects when administered two times in a monthly interval via ear wick according to the results of the CPTBCI-related quality of life assessment (Figure 4A) and CPTBCI foci surface area measurements (Figure 4B).

The treatment with PVRP resulted in a more significant improvement of the CPTBCI-related quality of life than the treatment with standard conservative methods (Figure 4A). Additionally, the CPTBCI foci surface areas decreased statistically significantly within 4 months in the PVRP group but not in the control group (Figure 4B). This trial may indicate long-term effects of PVRP-based treatment of CPTBCI according to the absence of symptoms in most patients for many months after the last (i.e., fourth) checkup of the treatment regime (Figure 4C). This corresponds to the long-term effects of platelet-rich preparations described by Altamura et al. (2020). No PVRP-related adverse events were detected in this trial, which is consistent with the findings of low adverse events rate in the meta-analysis of Wang et al. (2019). Therefore, PVRP is a safe method to treat CPTBCI.

The tolerable withdrawn blood volume (i.e., 18 ml) and the time required to prepare PVRP (i.e., *M* = 80 min, SD = 26 min) favor the treatment of other otorhinolaryngological diseases with PVRP on an outpatient or inpatient basis. To optimize platelet recovery and reduce withdrawn blood volume, theoretical models based on whole blood volume, hematocrit, and centrifugal conditions could be considered (Piao et al., 2017). Since there are heterogeneity and 10% replicability of PVRP preparation protocols, there is an urge to describe the PVRP and its preparation steps (Chahla et al., 2017). The thoroughly described sterile PVRP preparation protocol used in this trial yielded moderate platelet concentrations, which were identified as advantageous in the regeneration of PVRP as studied by DeLong et al. (2012).

Based on the CPTBCI treatment outcomes and identification of EVs in PVRP (Figure 5), it is believed that EVs, along with platelets, positively impact the regenerative properties of PVRP (Guo et al., 2017). It has been reported that platelet-derived EVs play significant roles in tissue regeneration, hemostasis, and immune response (Vozel et al., 2020b). A twofold mechanism could mediate these roles. EVs exhibit a larger surface-to-volume ratio than platelets and provide an extended catalytic area for biologically important reactions. By mediating interactions between cells, EVs carry signaling molecules for phenotypic transformation and serve as a cell mechanism to get rid of unwanted constituents (Ogorevc et al., 2014). In addition, platelet-derived EVs encapsulate principal growth factors from platelets (Guo et al., 2017) and are especially rich in TFG-β (transforming growth factor beta), bFGF (basic fibroblast growth factor), PDGF (platelet-derived growth factor), and VEGF (vascular endothelial growth factor) (Vozel et al., 2020b). Regenerative roles of platelet-derived EVs from PVRP were confirmed in a recent *in vitro* study by Otahal et al. (2020), where the induction of chondrogenic gene expression in chondrocytes and prevention of the release of proinflammatory cytokines were found. This study substantiates the regulatory roles of EVs in inflammation and metabolism of the cartilage extracellular matrix (Otahal et al., 2020). The above-listed properties of EVs could explain the regenerative, hemostatic, and immunological roles of PVRP in the treatment of CPTBCI. These roles could be regulated by choosing an appropriate PVRP activation procedure (i.e., exogenous or endogenous) and optimizing PVRP preparation protocol since the formation of EVs depends on *in vivo* or *ex vivo* platelet shedding (Vozel et al., 2020b).

There were no differences in baseline demographic, clinical characteristics, and COMQ-12 scores between groups, which confirm randomization (Table 1). To the best of our knowledge, there is no literature reporting the COMQ-12 scoring to assess CPTBCI-related quality of life. Moreover, the median COMQ-12 score of patients with CPTBCI (Mdn = 34) resembles the score of discharging COM without cholesteatoma (*M* = 33) as reported by Demir et al. (2020). For that reason, this trial confirms the significant socioeconomic and healthcare burden of CPTBCI (mainly chronically discharging radical mastoid cavity), comparable to COM (Khalil and Windle-Taylor, 2003). This burden, along with a long-term commitment to the patient and exhaustion of standard conservative treatment methods, is confirmed by the median time to treatment (i.e., 8.1 years) in our trial and was already reported by Dornhoffer et al. (2008). Analysis of performed procedures confirmed the exhaustion of surgical treatment methods. More radical surgical procedures (i.e., subtotal petrosectomy) would prevent serviceable hearing loss and not necessarily treat the CPTBCI (Yung, 2016). Additionally, the percentage of radical mastoidectomy performed to treat cholesteatoma (92%) and the predominance of male patients (82%) are consistent with the study of Khalil and Windle-Taylor (2003).

The results of risk factors of the patient (i.e., comorbidities, regular medicine intake, and harmful habits) are unique since there are no such data so far, to the best of our knowledge. However, there is no apparent predisposition to suffer from CPTBCI, according to the risk factor analysis. In contrast, two patients with coagulopathies (i.e., regular acetylsalicylic acid intake) were efficiently treated with PVRP. This also complements the study results by Smith et al. (2007), which reported that acetylsalicylic acid does not decrease the release of PDGF, which possesses essential regenerative roles of PVRP (Smith et al., 2007).

However, it is essential to balance the effects and limitations of PVRP-based therapies due to the high implicit hype of PVRP (Rachul et al., 2017). Since only 12% of patients were excluded from the trial after enrolment, the internal validity due to loss to follow-up is not at risk (Dettori et al., 2011). Issues related to the selection bias were avoided by the allocation concealment, and issues related to the performance bias were avoided by simple random allocation. Additionally, the blinded independent treatment outcome assessor measured the CPTBCI foci surface areas to minimize the detection bias. It is challenging to photograph with an otomicroscope under the same exposure at every checkup; therefore, CPTBCI foci surface area measurements could be biased.

Nonetheless, this objective measurement tool has reportedly not been utilized in inflammation area measurement so far. For that reason, it could provide the basis for further research in otology. Due to non-blind treatment allocation, the treatment outcome measured with COMQ-12 could present the detection bias. However, this disease-specific patient-reported outcome measure is possibly the most appropriate tool to assess CPTBCI-related quality of life, as already discussed. To the best of our knowledge, COMQ-12 has not been utilized to measure CPTBCI treatment outcome yet. In addition, to the best of our knowledge, the application of PVRP in the treatment of CPTBCI has been reported for the first time. Lastly, our trial is based on a relatively small sample collected in a single institution. CPTBCI is a rare disease that affects about 1.5% of patients with a radical mastoid cavity (Pareschi et al., 2019) or 0.8% after cholesteatoma surgery (Pareschi et al., 2019; Wilkie et al., 2019). Moreover, the risk of developing therapeutically resistant CPTBCI is even lower. Multicentric research could be conducted to overcome the limitation of a small sample size.

## Conclusion

Autologous PVRP represents a novel additional and successful treatment modality for a chronically discharging radical mastoid cavity when the surgical and standard conservative treatment methods have been exhausted. This disease is a type of CPTBCI and resembles COM without cholesteatoma in its impact on health-related quality of life, according to COMQ-12.

This trial contributes to further research on PVRP-based treatment for hearing loss (Stolle et al., 2018), facial nerve palsy (Sánchez et al., 2018), dysphonia (Cobden et al., 2016; Özgürsoy et al., 2018), dysphagia (Vidovic and Delling, 2017), anosmia (Yasak et al., 2018), soft-tissue defects (Eryılmaz et al., 2016; Tambella et al., 2018), or laryngotracheal reconstruction (Sahin Onder et al., 2020). In addition, sterile PVRP could be administered to the sterile human body areas. Since it is believed that platelets and EVs carry main benefits of PVRP, EV isolates from PVRP or various body fluids could be used to treat various diseases (Fais et al., 2016), including otological in the future (Kalinec et al., 2019).

## Data Availability Statement

The raw data supporting the conclusions of this article will be made available by the authors, without undue reservation.

## Ethics Statement

The studies involving human participants were reviewed and approved by National Medical Ethics Committee, Slovenia. The patients/participants provided their written informed consent to participate in this study.

## Author Contributions

DV contributed to the conceptualization, data curation, formal analysis, investigation, methodology, project administration, software, validation, visualization, and writing – original draft preparation, review, and editing. DB was involved in the data curation, funding acquisition, investigation, methodology, and writing – review and editing. MJ, ZJ, MP, and LP participated in the funding acquisition, investigation, and writing – review and editing. NS was involved in investigation and writing – review and editing. VK-I contributed to the conceptualization, funding acquisition, investigation, methodology, project administration, resources, software, supervision, validation, visualization, and writing – original draft preparation, review, and editing. SB contributed to the conceptualization, investigation, methodology, project administration, resources, software, supervision, visualization, and writing – original draft preparation, review, and editing. All authors contributed to the article and approved the submitted version.

## Conflict of Interest

The authors declare that the research was conducted in the absence of any commercial or financial relationships that could be construed as a potential conflict of interest.

## Abbreviations

COM: chronic otitis media
COMQ-12: chronic otitis media questionnaire-12
CPTBCI: chronic postoperative temporal bone cavity inflammation
EVs: extracellular vesicles
PDGF: platelet-derived growth factor
PRP: platelet-rich plasma
PVRP: platelet- and extracellular vesicle-rich plasma.

## References

[B1] Altamura S. A. Di Martino A. Andriolo L. Boffa A. Zaffagnini S. Cenacchi A. (2020). Platelet-Rich plasma for sport-active patients with knee osteoarthritis: limited return to sport. *Biomed. Res. Int.* 2020:8243865. 10.1155/2020/8243865 32076616 PMC7013341

[B2] Chahla J. Cinque M. E. Piuzzi N. S. Mannava S. Geeslin A. G. Murray I. R. (2017). A call for standardization in platelet-rich plasma preparation protocols and composition reporting: a systematic review of the clinical orthopaedic literature. *J. Bone. Joint. Surg. Am.* 99 1769–1779. 10.2106/JBJS.16.01374 29040132

[B3] Cobden S. B. Oztürk K. Duman S. Esen H. Aktan T. M. Avunduk M. C. (2016). Treatment of acute vocal fold injury with platelet-rich plasma. *J. Voice* 30 731–735. 10.1016/j.jvoice.2015.07.012 26292799

[B4] DeLong J. M. Russell R. P. Mazzocca A. D. (2012). Platelet-Rich plasma: the PAW classification system. *Arthroscopy* 28 998–1009. 10.1016/j.arthro.2012.04.148 22738751

[B5] Demir B. Sahin A. Binnetoglu A. Batman C. (2020). The utilization of chronic otitis media Questionnaire-12 in chronic otitis media with or without cholesteatoma. *Eur. Arch. Otorhinolaryngol.* 277 3037–3043. 10.1007/s00405-020-06030-7 32424496

[B6] Dettori J. R. (2011). Loss to follow-up. *Evid. Based Spine Care J.* 2 7–10. 10.1055/s-0030-1267080 22956930 PMC3427970

[B7] Dornhoffer J. L. Smith J. Richter G. Boeckmann J. (2008). Impact on quality of life after mastoid obliteration. *Laryngoscope* 118 1427–1432. 10.1097/MLG.0b013e318173dd7e 18475206

[B8] Eid A. M. Ebada H. A. El-Fattah A. M. A. Tawfik A. (2020). Platelet-rich fibrin: an autologous biomaterial for healing assistance of pharyngeal repair in total laryngectomy. *Eur. Arch. Otorhinolaryngol.* 278 463–470. 10.1007/s00405-020-06404-x 33009930

[B9] Eryılmaz A. Demirci B. Gunel C. Doger F. K. Yukselen O. Omurlu I. K. (2016). Can tissue adhesives and platelet-rich plasma prevent pharyngocutaneous fistula formation? *Auris Nasus Larynx* 43 62–67. 10.1016/j.anl.2015.06.012 26229017

[B10] Fais S. O’Driscoll L. Borras F. E. Buzas E. Camussi G. Cappello F. (2016). Evidence-Based clinical use of nanoscale extracellular vesicles in nanomedicine. *ACS Nano* 10 3886–3899. 10.1021/acsnano.5b08015 26978483

[B11] Gluth M. B. Tan B. Y. B. Santa Maria P. L. Atlas M. D. (2013). Unique microbiology of chronically unstable canal wall down tympanomastoid cavities: considerations for surgical revision. *J. Laryngol. Otol.* 127 458–462. 10.1017/S0022215113000583 23552343

[B12] Gökçe Kütük S. Özdaş T. (2019). The impact of platelet-rich plasma therapy on short-term postoperative outcomes of pediatric tonsillectomy patients. *Eur. Arch. Otorhinolaryngol.* 276 489–495. 10.1007/s00405-018-5211-1 30460402

[B13] Guo S.-C. Tao S.-C. Yin W.-J. Qi X. Yuan T. Zhang C.-Q. (2017). Exosomes derived from platelet-rich plasma promote the re-epithelization of chronic cutaneous wounds via activation of YAP in a diabetic rat model. *Theranostics* 7 81–96. 10.7150/thno.16803 28042318 PMC5196887

[B14] Henatsch D. Wesseling F. Briedé J. J. Stokroos R. J. (2015). Treatment of chronically infected open mastoid cavities with medical honey: a randomized controlled trial. *Otol. Neurotol.* 36 782–787. 10.1097/MAO.0000000000000728 25730446

[B15] Kalinec G. M. Gao L. Cohn W. Whitelegge J. P. Faull K. F. Kalinec F. (2019). Extracellular vesicles from auditory cells as nanocarriers for anti-inflammatory drugs and pro-resolving mediators. *Front. Cell Neurosci.* 13:530. 10.3389/fncel.2019.00530 31849615 PMC6895008

[B16] Khafagy Y. W. Abd Elfattah A. M. Moneir W. Salem E. H. (2018). Leukocyte- and platelet-rich fibrin: a new graft material in endoscopic repair of spontaneous CSF leaks. *Eur. Arch. Otorhinolaryngol.* 275 2245–2252. 10.1007/s00405-018-5048-7 29982939

[B17] Khalil H. S. Windle-Taylor P. C. (2003). Canal wall down mastoidectomy: a long term commitment to the outpatients? *BMC Ear Nose Throat Disord.* 3:1. 10.1186/1472-6815-3-1 12956889 PMC201024

[B18] Machin D. Fayers P. M. (2010). *Randomized Clinical Trials: Design, Practice and Reporting.* New Jersey: John Wiley & Sons Ltd, 95–111.

[B19] Ogorevc E. Hudoklin S. Veraniè P. Kralj-Iglič V. (2014). Extracellular vesicle-mediated transfer of membranous components from the highly malignant T24 urinary carcinoma cell line to the non-malignant RT4 urinary papilloma cell line. *Protoplasma* 251 699–702. 10.1007/s00709-013-0544-5 24019014

[B20] Otahal A. Kramer K. Kuten-Pella O. Weiss R. Stotter C. Lacza Z. (2020). Characterization and chondroprotective effects of extracellular vesicles from plasma- and serum-based autologous blood-derived products for osteoarthritis therapy. *Front. Bioeng. Biotechnol.* 8:584050. 10.3389/fbioe.2020.584050 33102466 PMC7546339

[B21] Özgürsoy S. K. Tunçkaşık F. Tunçkaşık M. E. Akıncıoğlu E. Doğan H. Beriat G. K. (2018). Histopathologic evaluation of hyaluronic acid and plasma-rich platelet injection into rabbit vocal cords: an experimental study. *Turk Arch. Otorhinolaryngol.* 56 30–35. 10.5152/tao.2018.2942 29988271 PMC6017207

[B22] Pachito D. V. Latorraca C. de Oc Riera R. (2019). Efficacy of platelet-rich plasma for non-transfusion use: overview of systematic reviews. *Int. J. Clin. Pract.* 73:e13402. 10.1111/ijcp.13402 31408240

[B23] Pareschi R. Lepera D. Nucci R. (2019). Canal wall down approach for tympano-mastoid cholesteatoma: long-term results and prognostic factors. *Acta Otorhinolaryngol. Ital.* 39 122–129. 10.14639/0392-100X-2237 31097831 PMC6522862

[B24] Phillips J. S. Haggard M. Yung M. (2014). A new health-related quality of life measure for active chronic otitis media (COMQ-12): development and initial validation. *Otol. Neurotol.* 35 454–458. 10.1097/MAO.0000000000000205 24518406

[B25] Piao L. Park H. Jo C. H. (2017). Theoretical prediction and validation of cell recovery rates in preparing platelet-rich plasma through a centrifugation. *PLoS One* 12:e0187509. 10.1371/journal.pone.0187509 29095890 PMC5667898

[B26] Rachul C. Rasko J. E. J. Caulfield T. (2017). Implicit hype? representations of platelet rich plasma in the news media. *PLoS One* 12:e0182496. 10.1371/journal.pone.0182496 28792974 PMC5549909

[B27] Sahin Onder S. Sahin Yilmaz A. Erkmen B. Topal C. S. Gergin O. Canpolat M. S. (2020). Platelet-rich plasma for laryngotracheal reconstruction: an experimental study. *Eur. Arch. Otorhinolaryngol.* 277 3103–3109. 10.1007/s00405-020-06091-8 32476045

[B28] Sánchez M. Garate A. Bilbao A. M. Oraa J. Yangüela F. Sánchez P. (2018). *Platelet-Rich Plasma for Injured Peripheral Nerves: Biological Repair Process and Clinical Application Guidelines. Demystifying Polyneuropathy - Recent Advances and New Directions.* Available online at: https://www.intechopen.com/books/demystifying-polyneuropathy-recent-advances-and-new-directions/platelet-rich-plasma-for-injured-peripheral-nerves-biological-repair-process-and-clinical-applicatio (Accessed September 25, 2019)

[B29] Schulz K. F. Altman D. G. Moher D. Consort Group (2010). CONSORT 2010 statement: updated guidelines for reporting parallel group randomized trials. *Ann. Intern. Med.* 152 726–732. 20335313 10.7326/0003-4819-152-11-201006010-00232

[B30] Smith C. W. Binford R. S. Holt D. W. Webb D. P. (2007). Quality assessment of platelet rich plasma during anti-platelet therapy. *Perfusion* 22 41–50. 10.1177/0267659107077950 17633134

[B31] Stolle M. Schulze J. Roemer A. Lenarz T. Durisin M. Warnecke A. (2018). Human plasma rich in growth factors improves survival and neurite outgrowth of spiral ganglion neurons in vitro. *Tissue Eng. Part A* 24 493–501. 10.1089/ten.TEA.2017.0120 28610547

[B32] Šuštar V. Bedina-Zavec A. Štukelj R. Frank M. Bobojević G. Janša R. (2011). Nanoparticles isolated from blood: a reflection of vesiculability of blood cells during the isolation process. *Int. J. Nanomed.* 6 2737–2748. 10.2147/IJN.S24537 22128248 PMC3225219

[B33] Tambella A. M. Attili A. R. Dupré G. Cantalamessa A. Martin S. Cuteri V. (2018). Platelet-rich plasma to treat experimentally-induced skin wounds in animals: a systematic review and meta-analysis. *PLoS One* 13:e0191093. 10.1371/journal.pone.0191093 29324848 PMC5764374

[B34] Tao S.-C. Guo S.-C. Zhang C.-Q. (2017). Platelet-derived extracellular vesicles: an emerging therapeutic approach. *Int. J. Biol. Sci.* 13 828–834. 10.7150/ijbs.19776 28808416 PMC5555101

[B35] Vidovic A. Delling U. (2017). Aryepiglottic fold augmentation as treatment for late-onset dysphagia following surgical treatment of recurrent laryngeal neuropathy. *Tierarztl Prax Ausg G Grosstiere Nutztiere* 45 219–225. 10.15653/TPG-160712 28745776

[B36] Vozel D. Božič D. Jeran M. Jan Z. Pajnič M. Paðen L. (2020a). “The role of platelet- and extracellular vesicle-rich plasma in the treatment of chronic postoperative temporal bone cavity inflammation: a randomized controlled clinical trial,” in *Socratic Lectures: 3rd International Minisymposium: Peer Reviewed Proceedings*, (Ljubljana: Faculty of Health Sciences, University of Ljubljana, Slovenia).

[B37] Vozel D. Božič D. Jeran M. Jan Z. Pajnič M. Paðen L. (2020b). “Treatment with platelet- and extracellular vesicle-rich plasma in otorhinolaryngology-a review and future perspectives,” in *Advances in Biomembranes and Lipid Self-Assembly*, eds Bongiovanni A. Pocsfalvi G. Manno M. Kralj-Igli? V. (Amsterdam: Elsevier), 10.1016/bs.abl.2020.05.003

[B38] Vozel D. Steiner N. Božanić Urbančič N. Mladenov D. Battelino S. (2020c). Slovenian cross-cultural adaptation and validation of health-related quality of life measures for chronic otitis media (COMQ-12), vertigo (DHI, NVI) and tinnitus (THI). *Zdr Varst* 59 120–127. 10.2478/sjph-2020-0016 32952712 PMC7478096

[B39] Wang C. Xu M. Guo W. Wang Y. Zhao S. Zhong L. (2019). Clinical efficacy and safety of platelet-rich plasma in arthroscopic full-thickness rotator cuff repair: a meta-analysis. *PLoS One* 14:e0220392. 10.1371/journal.pone.0220392 31356630 PMC6663026

[B40] Wilkie M. D. Chudek D. Webb C. J. Panarese A. Banhegyi G. (2019). Canal wall down mastoidectomy with obliteration versus canal wall up mastoidectomy in primary cholesteatoma surgery. *J. Laryngol. Otol.* 133 1074–1078. 10.1017/S0022215119002408 31735175

[B41] Yáñez-Mó M. Siljander P. R.-M. Andreu Z. Zavec A. B. Borràs F. E. Buzas E. I. (2015). Biological properties of extracellular vesicles and their physiological functions. *J. Extracell Vesicles* 4:27066. 10.3402/jev.v4.27066 25979354 PMC4433489

[B42] Yasak A. G. Yigit O. Araz Server E. Durna Dastan S. Gul M. (2018). The effectiveness of platelet-rich plasma in an anosmia-induced mice model. *Laryngoscope* 128 E157–E162. 10.1002/lary.27029 29243256

[B43] Yung M. (2016). The use of temporoparietal fascial flap to eliminate wound breakdown in subtotal petrosectomy for chronic discharging ears. *Otol. Neurotol.* 37 248–251. 10.1097/MAO.0000000000000959 26808557

