# Supplementary material for: Autologous Platelet- and Extracellular Vesicle-Rich Plasma Is an Effective Treatment Modality for Chronic Postoperative Temporal Bone Cavity Inflammation: Randomized Controlled Clinical Trial
Source: Front Bioeng Biotechnol. 2021 Jul 7;9:677541. doi: 10.3389/fbioe.2021.677541 (PMC8294456; doi:10.3389/fbioe.2021.677541)
Supplement: Supplementary file 1 [file Table_1.DOCX]

Supplementary Material 1

# Randomization process

Patients with bilateral chronic postoperative temporal bone cavity inflammation (CPTBCI) were allocated to the intervention A (i.e., control group: treatment with standard conservative methods) as the treatment of bilateral CPTBCI with platelet- and extracellular vesicle rich plasma (i.e., PVRP) would require the twice of the blood volume needed as the preparation of PVRP for the treatment of unilateral CPTBCI. The treatment outcome assessment with chronic otitis media questionnaire 12 (COMQ-12) could not be applied if the trial would be planned as a split-ear trial (i.e., each CPTBCI allocated to each intervention). The remaining patients, i.e., patients with unilateral CPTBCI, were allocated to intervention A (i.e., control group) or B (i.e., treatment with PVRP or PVRP group) by simple random allocation. Therefore, patients with bilateral CPTBCI were not included in intervention B. Simple random allocation was performed by a researcher (VK-I) who was not informed about the patient's data. The process was performed according to the literature [1] in the following steps:

1. The names of all enrolled patients with unilateral CPTBCI were placed in separate sealed envelopes so that their identities were hidden.
2. We have determined that the assigned even numbers (including the number zero) are later allocated to intervention A and odd numbers to intervention B.
3. The sealed envelopes were arranged sequentially on the table.
4. A sequence of random numbers was chosen from the table of random numbers (Figure) for each envelope [2].
5. We wrote the number from left to right in the same order as in the table of random numbers on each envelope.
6. We allocated intervention A to even numbers (including the number 0) on the envelopes and intervention B to odd numbers.
7. The envelopes were opened.


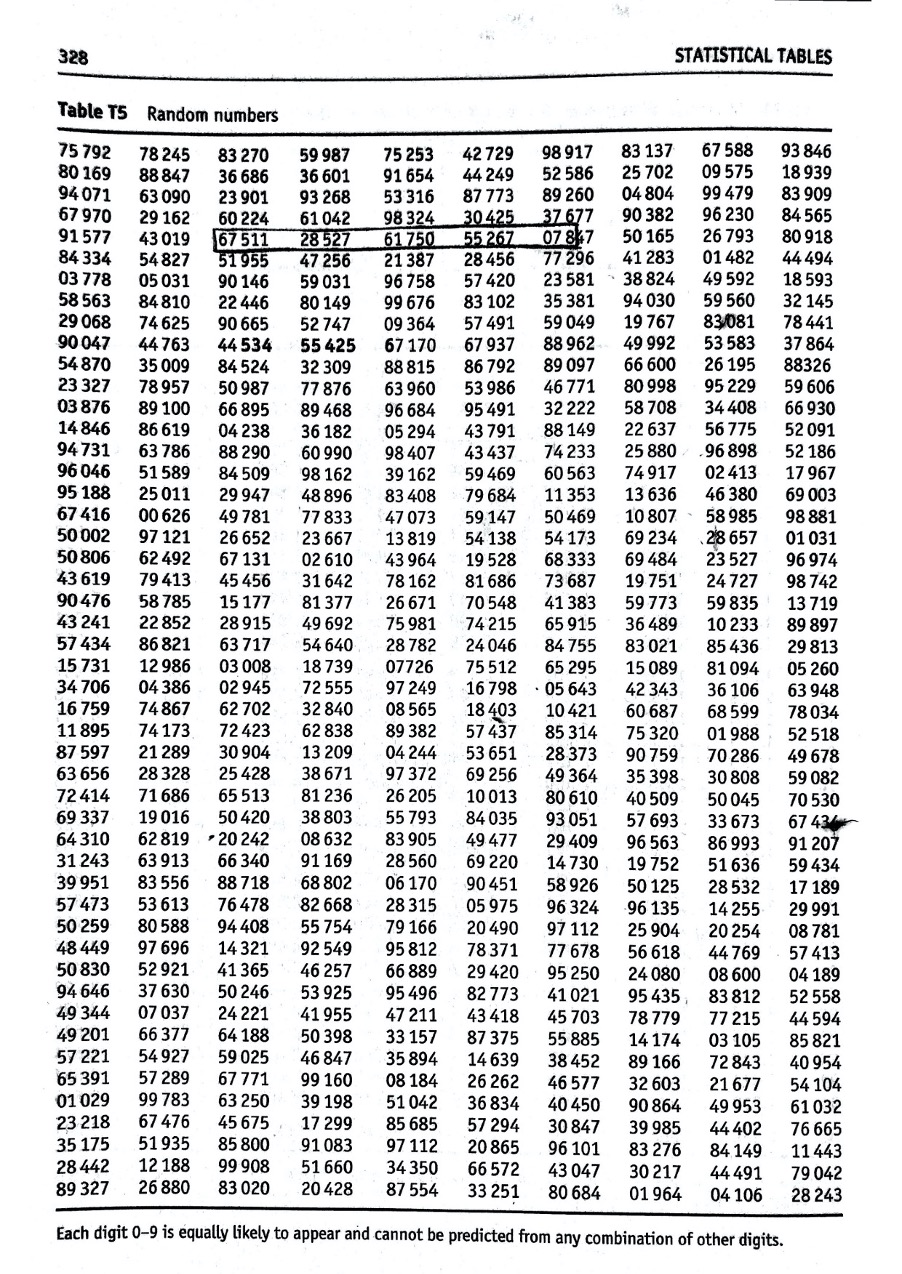


**Supplementary Figure:** Table of random numbers with the chosen number sequence, used in randomization process [2]. The series of 25 numbers were selected from the table of random numbers. These numbers were sequentially written on the sealed envelopes.

## References

1. Machin D, Fayers PM (2010) Randomization. In: Randomized Clinical Trials: Design, Practice and Reporting. John Wiley & Sons Ltd, New Jersey, pp 95–111

2. Machin D, Fayers PM (2010) Statistical Tables. In: Randomized Clinical Trials: Design, Practice and Reporting. John Wiley & Sons Ltd, New Jersey, p 328
