# Supplementary material for: Autologous Platelet- and Extracellular Vesicle-Rich Plasma Is an Effective Treatment Modality for Chronic Postoperative Temporal Bone Cavity Inflammation: Randomized Controlled Clinical Trial
Source: Front Bioeng Biotechnol. 2021 Jul 7;9:677541. doi: 10.3389/fbioe.2021.677541 (PMC8294456; doi:10.3389/fbioe.2021.677541)
Supplement: Supplementary file 2 [file Table_2.DOCX]

Supplementary Material 2

# Anatomical classification scheme


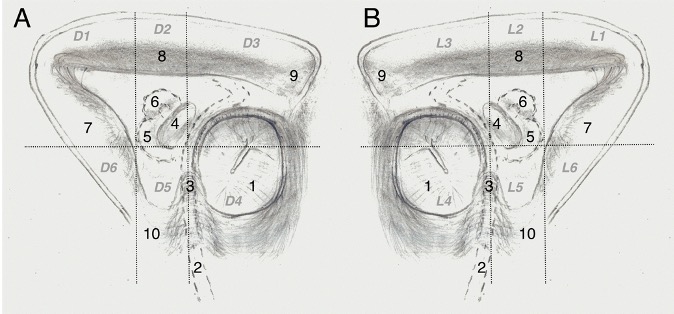


**Supplementary Figure:** An illustration of postoperative temporal bone cavities, i.e., radical cavities. Illustration of right (A) and left (B) postoperative temporal bone cavity with areas (D1-D6, L1-L6) and marked relevant anatomical landmarks: *pars tensa* of the tympanic membrane (1), the base of ablated posterior external auditory canal wall (2), facial nerve ridge (3), facial nerve (dashed curves), lateral (4), posterior (5) and the superior semicircular canal (6), sigmoid sinus (7), middle cranial fossa dura (8), zygomatic root (9), mastoid apex (10)
