# Supplementary material for: Autologous Platelet- and Extracellular Vesicle-Rich Plasma Is an Effective Treatment Modality for Chronic Postoperative Temporal Bone Cavity Inflammation: Randomized Controlled Clinical Trial
Source: Front Bioeng Biotechnol. 2021 Jul 7;9:677541. doi: 10.3389/fbioe.2021.677541 (PMC8294456; doi:10.3389/fbioe.2021.677541)
Supplement: Supplementary file 3 [file Table_3.DOCX]

Supplementary Material 3

## Scanning electron microscopy procedure of platelet- and extracellular vesicle-rich plasma

Two PVRP samples were prepared for analysis by scanning electron microscopy by a slightly modified procedure adopted from Lešer et al. (2007) (36). Samples were first fixed overnight at 4 °C in a modified Karnovsky fixative (2,5% glutaraldehyde (SPI-Chem, West Chester, USA); 0,4% formaldehyde (Sigma-Aldrich, Steinheim, Germany) in 300mM phosphate buffer pH 7.4 (137 mM NaCl, 2,68 mM KCl, 10,14 mM Na_2_HPO_4,_ 1,84 mM KH_2_PO_4_ (all chemical were purchased from Sigma-Aldrich, Steinheim, Germany)). The next day, we performed post-fixation with OsO_4_ (SPI-Chem, West Chester, USA). Primary fixatives were removed in three steps of washing with phosphate buffer (in each step, the previous solution was replaced with fresh buffer, and the samples were incubated for 10 minutes). The washed samples were then incubated in 2% OsO_4_ for one hour. After incubation, the osmium was removed, and the samples were washed three times with distilled water (incubation time 10 minutes in each step), incubated for 15 minutes in a saturated aqueous solution of thiocarbohydrazide (Sigma-Aldrich, Steinheim, Germany), rewashed three times with distilled water and treated once again in 2% OsO_4_. The osmium was then removed, the samples were washed three times with distilled water and gradually dehydrated in increasing concentrations of ethanol (Emplura, Merck, Darmstadt, Germany; the samples were incubated 10 minutes in 30%, 50%, 70%, 80%, 90%, and absolute ethanol, the step with absolute ethanol was repeated three times) and then gradually replaced with hexamethyldisilazane (HMDS, Sigma-Aldrich, Steinheim, Germany). The samples were incubated for 10 minutes in 30% and 50% HMDS mixture with absolute ethanol and then in pure HMDS and finally, air-dried. The dried samples were dusted with a mix of gold and palladium and analyzed with a scanning electron microscope (JSM-6500F, JEOL Ltd., Tokyo, Japan) (36).


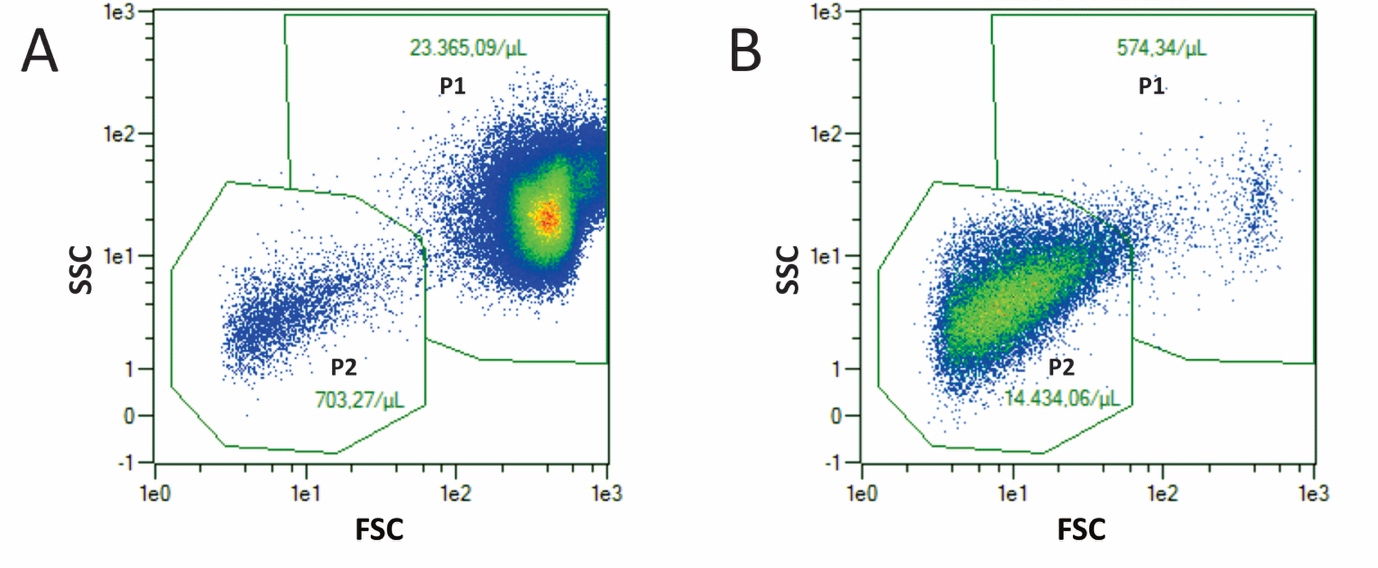
**Supplementary Figure:** Examples of scattering plots obtained by flow cytometry for blood (A) and a PVRP sample (B). Gates P1(mainly erythrocytes and leucocytes) and P2 (mainly platelets and large extracellular vesicles) are presented to illustrate the clouds' location. In the case of blood, the number of leucocytes in P1 is negligible compared to erythrocytes, while in the case of PVRP, the ratio becomes substantially shifted in favor of leukocytes. Event counts given in the presented plots must be considered concerning the dilution factor (1:200 for the blood and 1:50 for the PVRP). PVRP – platelet- and extracellular vesicle-rich plasma; SSC – side scatter signal; FSC – forward scatter signal. The Colour scale from blue to red corresponds to increasing event density.
