# Supplementary material for: Autologous Platelet- and Extracellular Vesicle-Rich Plasma Is an Effective Treatment Modality for Chronic Postoperative Temporal Bone Cavity Inflammation: Randomized Controlled Clinical Trial
Source: Front Bioeng Biotechnol. 2021 Jul 7;9:677541. doi: 10.3389/fbioe.2021.677541 (PMC8294456; doi:10.3389/fbioe.2021.677541)
Supplement: Supplementary file 4 [file Table_4.docx]

Supplementary Material 4

# Chemicals used for analyses

Chemicals used for the analysis of platelet- and extracellular vesicle-rich plasma (PVRP) and blood:

- Buffer for flow cytometer (MACSQuant Running Buffer, ref.: 130-092-747, lot: various, Miltenyi Biotec B.V. & Co. KG, Germany).
- Phosphate buffer (pH 7,4), prepared from:
- Phosphate-citrate buffer (pH 7,4), prepared from:
  - phosphate buffer;
    - 10,9 mM Na_3_C_6_H_5_O_7_ × *2* H_2_O (Carlo Erba Reagents, CAS: 6132-04-3, Rodano, Italy)

Then filtered through filters Chromafil RC-20/25 (ref.: 729030, lot: 9.155, Macherey-Nagel GmbH, Germany) with 0.2 microns permeability.

# Sterility analyses of platelet- and extracellular vesicle-rich plasma

PVRP was prepared for sterility analysis using the preparation protocol described in the main text from volunteers, aged over 18 years and without CPTBCI and associated diseases and signs of infection. Two PVRP units were transferred to a microbiological medium (BD BACTEC Peds Plus / F Culture Vials (plastic), ref: 449456142765, lot: 9186899, Becton Dickinson, USA), which is also intended for culturing and isolating bacteria and fungi from umbilical cord blood. The preparation of PVRP for sterility analysis took place together with the preparation of PBTZV for patients with CVD. The sterility analysis of PBTZV was performed at the Institute of Microbiology and Immunology, University of Ljubljana, Faculty of Medicine. The samples were processed on a Bactec FX system (BectonDickinson, USA). Samples of vials with detected microbes were inoculated onto bacteriological media (Columbia agar and chocolate agar) and grown colonies of bacteria or fungi were identified by MALDI-TOF MS mass spectrometry (Bruker Corporation, USA). Sensitivity to antibiotics or antifungals was determined for the identified microbes. Specialists in clinical microbiology participated in the selection of sterility analysis procedures.
