# Supplementary material for: Autologous Platelet- and Extracellular Vesicle-Rich Plasma Is an Effective Treatment Modality for Chronic Postoperative Temporal Bone Cavity Inflammation: Randomized Controlled Clinical Trial
Source: Front Bioeng Biotechnol. 2021 Jul 7;9:677541. doi: 10.3389/fbioe.2021.677541 (PMC8294456; doi:10.3389/fbioe.2021.677541)
Supplement: Supplementary file 5 [file Table_5.DOCX]

Supplementary Material 5

# Statistical analysis

## Statistical analysis of continuous variables

To compare the differences between the two groups of independent continuous numerical variables (e.g., the comparison between PVRP and the control group), we first determined the outliers in each group. Outliers were defined as values ​​greater than 1.5 times the box's length (i.e., the length of the boxplot without the whiskers). We then determined the distribution in each group using the Shapiro-Wilk test. A p-value (*p*) <0.05, the distribution was non-normal. If there were no outliers in groups and the distribution was normal, we used the t-test for independent samples. Levene's test checked the homogeneity of the variances between the groups. If at *p*<0.05 the groups' variance was different (i.e., heterogeneous), we used a t-test for independent samples, adapted for the case of violation of the homogeneity of variances. For homogeneity of variances, we used a normal t-test for independent samples. Descriptive t-test statistics for independent samples were described by mean (*M*), standard deviation (*SD*), and 95% confidence interval (95% CI), and the statistical significance was defined by the value of the test statistic (*t*) at degrees of freedom (listed in parentheses) and with *p*.

If outliers and/or a non-normal distribution were present in at least one of the two independent groups with continuous numerical variables, the groups were compared with the Mann-Whitney U-test. Data transformation or elimination of outliers were not applied due to the significant impact on results. We first compared the shape of both groups' distributions; if they were similar, we compared the medians with the Mann-Whitney U-test. Descriptive statistics of the Mann-Whitney U-test were presented with median (*Mdn*) and statistical significance with the *p*.

To compare the differences between four groups of dependent and two groups of independent continuous numerical variables (e.g., comparison of COMQ-12 sum scores between the four check-ups and at the same time between PVRP and control group), we checked the assumption for performing a two-way mixed analysis of variance (two-way mixed ANOVA). We first determined the outliers, the shape of the distribution, and the homogeneity of each group's variances. In the absence of outliers, normal distribution, and homogeneity of variances, we tested the homogeneity of covariances with the Box's M-test. At *p*>0.05, the covariances of groups were homogeneous, so we determined the sphericity with Mauchly's test. At *p*>0.05 with Mauchly's test, a two-way mixed ANOVA with *post-hoc* testing was performed. Descriptive statistics of two-way mixed ANOVA were presented with a mean (*M*) and standard error (*SE*), and the statistical significance with the value of test statistics *F* at degrees of freedom (in parentheses), with *p* and with effect size measure (partial *η^2^*) at *p*>0.05.

To compare the differences between at least four groups of dependent continuous numerical variables (e.g., comparing the CPTBCI focus surface area between the four check-ups), we first checked the assumptions for using one-way repeated measures ANOVA. Outliers, the shape of distributions, and sphericity were determined in all groups. When meeting the assumption of the sphericity, we performed a standard one-way repeated measures ANOVA. When the sphericity was violated, a Greenhouse-Geisser correction with the epsilon (*ε*) value was applied for the one-way repeated measures ANOVA. We then analyzed the differences between the pairs of groups by *post-hoc* analysis using the Bonferroni correction. Descriptive statistics of one-way repeated measures ANOVA were described with a mean (*M*), standard deviation (*SD*) and 95% confidence interval (95% CI), and statistical significance with the value of test statistics *F* at degrees of freedom (in parentheses), with *p* and with a measure of the size of the effect (partial *ω^2^*) at *p*>0.05.

## Statistical analysis of dichotomous variables

To compare the differences between the two groups of independent dichotomous variables (e.g., gender differences between PVRP and control group), we used Fisher's exact test for samples of size <5 and the *χ2* test of homogeneity for sample size >5. Descriptive statistics of the dichotomous variable analysis were presented with a percentage and statistical significance with a *p*.
