# Supplementary material for: Autologous Platelet- and Extracellular Vesicle-Rich Plasma Is an Effective Treatment Modality for Chronic Postoperative Temporal Bone Cavity Inflammation: Randomized Controlled Clinical Trial
Source: Front Bioeng Biotechnol. 2021 Jul 7;9:677541. doi: 10.3389/fbioe.2021.677541 (PMC8294456; doi:10.3389/fbioe.2021.677541)
Supplement: Supplementary file 7 [file Table_7.DOCX]

Supplementary Material 7

# Tables of patient's baseline characteristics

**Supplementary Table 1:** Baseline demographic and clinical characteristics of 11 patients treated with platelet- and extracellular vesicle-rich plasma. Remarks: M – male; F – female; 0 – the absence of the condition; the 1 – presence of the condition; ^a^ – arterial hypertension; ^b^ – dyslipidemia; ^c^ – gastroesophageal reflux; ^d^ – arterial thrombosis; ^e^ – allergic dermatitis; ^f^ – allergic rhinitis; ^g^ – retinal detachment; ^h^ – asthma; ^i^ – benign prostatic hyperplasia; ^j^ – suffered cerebrovascular insult; EAC – external auditory canal; CWD – »canal-wall down« or radical mastoidectomy; Time from otosurgery – the time from the last surgery required for the underlying disease of the temporal bone to the trial's first check-up; Time to therapy – the time from onset of problems due to the chronic postoperative temporal bone cavity inflammation to the trial's first check-up; NA – missing data.

| ID | Gender | Age | Other diseases | Allergies | Coagulation disorder | Smoking | Alcohol | Regular medicine intake | Underlying  temporal bone cavity  disease | Type of otosurgery | Time from otosurgery (days) | Timefrom otosurgery (years) | Time to therapy (days) | Time to therapy (years) |
| --- | --- | --- | --- | --- | --- | --- | --- | --- | --- | --- | --- | --- | --- | --- |
| 1 | M | 72 | 1^a-b-c,d^ | 1 | 1 | 0 | 1 | 1 | EAC cholesteatoma | CWD | 457 | 1.3 | 294 | 0.8 |
| 2 | F | 39 | 1^e,f^ | 1 | 0 | 0 | 0 | 0 | middle ear cholesteatoma | CWD | 174 | 0.5 | 91 | 0.2 |
| 3 | M | 24 | 0 | 0 | 0 | 0 | 1 | 0 | middle ear cholesteatoma | CWD | 3964 | 10.9 | 1577 | 4.3 |
| 4 | M | 34 | 0 | 0 | 0 | 0 | 1 | 0 | middle ear cholesteatoma | CWD | NA | NA | 3185 | 8.7 |
| 5 | F | 55 | 1^g^ | 1 | 0 | 0 | 1 | 0 | middle ear cholesteatoma | CWD | 212 | 0.6 | 58 | 0.2 |
| 6 | M | 26 | 1^h^ | 1 | 0 | 1 | 1 | 0 | middle ear cholesteatoma | CWD | 5957 | 16.3 | 2893 | 7.9 |
| 7 | M | 76 | 1^i,j^ | 0 | 1 | 0 | 1 | 1 | middle ear cholesteatoma | CWD | 8330 | 22.8 | 762 | 2.1 |
| 8 | M | 44 | 0 | 0 | 0 | 1 | 1 | 0 | middle ear cholesteatoma | CWD | 2778 | 7.6 | 2688 | 7.4 |
| 9 | M | 45 | 0 | 1 | 0 | 0 | 1 | 0 | middle ear cholesteatoma | CWD | 5323 | 14.6 | 2969 | 8.1 |
| 10 | M | 38 | 0 | 0 | 0 | 0 | 1 | 0 | middle ear cholesteatoma | CWD | 8379 | 23.0 | 3290 | 9.0 |
| 11 | M | 68 | 1^a^ | 0 | 0 | 0 | 1 | 1 | middle ear cholesteatoma | CWD | 21570 | 59.1 | 2752 | 7.5 |

| ID | Gender | Age | Other diseases | Allergies | Coagulation disorder | Smoking | Alcohol | Regular medicine intake | Underlying  temporal bone cavity  disease | Type of otosurgery | Time from otosurgery (days) | Timefrom otosurgery (years) | Time to therapy (days) | Time to therapy (years) |
| --- | --- | --- | --- | --- | --- | --- | --- | --- | --- | --- | --- | --- | --- | --- |
| 12 | F | 31 | 1^k,l^ | 0 | 0 | 1 | 0 | 1 | middle ear cholesteatoma | CWD | 1101 | 3.0 | 846 | 2.3 |
| 13 | M | 66 | 1^a,b^ | 0 | 0 | 1 | 1 | 1 | middle ear cholesteatoma | CWD | NA | NA | 3172 | 8.7 |
| 14 | M | 66 | 1^m^ | 0 | 0 | 0 | 1 | 1 | EAC SCC | SP | 5720 | 15.7 | 2086 | 5.7 |
| 15L | F | 39 | 0 | 1 | 0 | 1 | 0 | 0 | middle ear cholesteatoma | CWD | NA | NA | 2666 | 7.3 |
| 15R |  |  |  |  |  |  |  |  | middle ear cholesteatoma | CWD | NA | NA | 3366 | 9.2 |
| 16 | M | 73 | 1^h,i^ | 1 | 0 | 1 | 0 | 1 | middle ear cholesteatoma | CWD | 8358 | 22.9 | 2926 | 8.0 |
| 17 | M | 68 | 1^a,b,n,o,p^ | 0 | 1 | 0 | 1 | 1 | middle ear cholesteatoma | CWD | 12740 | 34.9 | 260 | 0.7 |
| 18 | M | 21 | 0 | 1 | 0 | 0 | 1 | 0 | middle ear cholesteatoma | CWD | 3388 | 9.3 | 1733 | 4.7 |
| 19 | M | 89 | 0 | 0 | 0 | 0 | 0 | 0 | middle ear cholesteatoma | CWD | 4727 | 13.0 | 3297 | 9.0 |
| 20 | M | 18 | 0 | 0 | 0 | 0 | 1 | 0 | middle ear cholesteatoma | CWD | 1249 | 3.4 | 3257 | 8.9 |
| 21L | M | 69 | 1^a,b,q,r^ | 1 | 0 | 0 | 1 | 1 | middle ear cholesteatoma | CWD | NA | NA | 3255 | 8.9 |
| 21R |  |  |  |  |  |  |  |  | middle ear cholesteatoma | CWD | NA | NA | 3255 | 8.9 |
| 22 | M | 30 | 0 | 0 | 0 | 1 | 1 | 0 | middle ear cholesteatoma | CWD | 9510 | 26.1 | 3219 | 8.8 |

**Supplementary Table 2:** Baseline demographic and clinical characteristics of 11 patients treated with standard conservative measures. Patients 15 and 21 had bilateral involvement (KVPVS). L – left temporal bone; R – right temporal bone. M – male; F – female 0 – the absence of the condition; 1 – the presence of the condition; ^a^ – arterial hypertension; ^b^ – dyslipidemia; ^h^ – asthma; ^i^ – benign prostatic hyperplasia; ^k^ – depression; ^l^ – suffered head and spleen injury; ^m^ – arthralgias; ^n^ – diabetes; ^o^  – hypothyroidism; ^p^ – hypersomnolence; ^q^ – suffered bowel cancer; ^r^ – suffered sepsis; PCC – squamous-cell carcinoma; CWD – »canal-wall down« or radical mastoidectomy; EAC SCC– external auditory canal squamous cell carcinoma; SP – subtotal petrosectomy; Time from otosurgery – the time from the last surgery required for the underlying disease of the temporal bone to the trial's first check-up; Time to therapy – the time from onset of problems due to the chronic postoperative temporal bone cavity inflammation to the trial's first check-up; NA – missing data.
