# Supplementary material for: Autologous Platelet- and Extracellular Vesicle-Rich Plasma Is an Effective Treatment Modality for Chronic Postoperative Temporal Bone Cavity Inflammation: Randomized Controlled Clinical Trial
Source: Front Bioeng Biotechnol. 2021 Jul 7;9:677541. doi: 10.3389/fbioe.2021.677541 (PMC8294456; doi:10.3389/fbioe.2021.677541)
Supplement: Supplementary file 8 [file Table_8.DOCX]

Supplementary Material 8

# Platelet- and extracellular vesicle-rich plasma preparation analyses

**Supplementary Table:** Data regarding the preparation and composition of platelet- and extracellular vesicle-rich plasma (PVRP) for patients with chronic postoperative temporal bone cavity inflammation treated with two PVRP administrations. Time to prepare PVRP refers to the time from venipuncture to resuspension of PVRP. The volume of plasma and volume of PVRP are totaled as they represent the preparation of two PVRP units. Therefore, the volume of preparation of one unit should be divided by two. Platelet concentrations in blood ([Platelets_BLOOD_]) and in PVRP ([Platelets_PVRP_]) were determined by flow cytometry. From these data, the percentage of platelets in PVRP (% of platelets) was calculated. NA – missing data.

|  | ID | Time to prepare PVRP (min) | The volume of plasma (mL) | The volume of PVRP (mL) | [Platelets_BLOOD_]  (×10^9^/L) | [Platelets_PVRP_]  (×10^9^/L) | % of platelets |
| --- | --- | --- | --- | --- | --- | --- | --- |
| 1^st^ application of PVRP | 1 | 65 | 3.8 | 1.9 | 164 | 419 | 256 |
|  | 2 | 100 | 4.5 | 2.3 | 288 | 389 | 135 |
|  | 3 | 95 | 2.5 | 1.3 | 111 | 515 | 464 |
|  | 4 | 51 | 4.0 | 2.0 | 245 | 553 | 226 |
|  | 5 | 68 | 6.8 | 3.4 | 280 | 512 | 183 |
|  | 6 | 65 | 6.6 | 3.3 | 223 | 385 | 173 |
|  | 7 | 66 | 5.0 | 1.3 | 167 | 274 | 164 |
|  | 8 | 73 | 3.7 | 1.9 | 155 | 498 | 321 |
|  | 9 | 70 | 3.0 | 1.5 | 118 | 868 | 737 |
|  | 10 | 70 | 3.8 | 1.9 | 185 | 491 | 265 |
|  | 11 | 49 | 2.8 | 1.4 | 308 | 685 | 222 |
| 2^nd^ application of PVRP | 1 | 71 | 5.0 | 2.5 | 297 | 434 | 146 |
|  | 2 | 132 | 5.3 | 2.7 | 185 | 320 | 173 |
|  | 3 | 75 | 3.0 | 1.5 | 129 | 235 | 182 |
|  | 4 | 55 | 3.8 | 1.9 | 199 | 831 | 416 |
|  | 5 | 127 | 6.2 | 3.1 | 229 | 550 | 241 |
|  | 6 | 120 | 6.9 | 3.5 | 231 | 722 | 312 |
|  | 7 | 81 | 4.1 | 2.1 | 200 | 443 | 221 |
|  | 8 | 115 | 2.7 | 1.7 | 108 | NA | NA |
|  | 9 | 98 | 4.8 | 1.8 | 259 | 134 | 52 |
|  | 10 | 63 | 2.4 | 1.2 | 155 | 605 | 390 |
|  | 11 | 42 | 3,2 | 1.6 | 141 | 729 | 518 |
