# Supplementary material for: Autologous Platelet- and Extracellular Vesicle-Rich Plasma Is an Effective Treatment Modality for Chronic Postoperative Temporal Bone Cavity Inflammation: Randomized Controlled Clinical Trial
Source: Front Bioeng Biotechnol. 2021 Jul 7;9:677541. doi: 10.3389/fbioe.2021.677541 (PMC8294456; doi:10.3389/fbioe.2021.677541)
Supplement: Supplementary file 9 [file Table_9.DOCX]

Supplementary Material 9

# The intervals between check-ups and results of the treatment outcome measures

**Supplementary Table:** The intervals between the check-ups (1.-2., 2.-3. and 3.-4.), the proportions of the chronic postoperative temporal bone cavity inflammation (CPTBCI) foci surface areas, and the chronic otitis media questionnaire 12 (COMQ-12) sum scores at four check-ups. IDs 1-11 denote patients treated with platelet- and extracellular vesicle-rich plasma, and 12-22 indicate patients treated with standard conservative measures. Patients 15 and 21 had bilateral involvement. L – left temporal bone; R – right temporal bone.

|  | Interval between check-ups  (days) | | | CPTBCI focus surface area  (%) | | | | COMQ-12 sum score | | | |
| --- | --- | --- | --- | --- | --- | --- | --- | --- | --- | --- | --- |
| ID | *1.–2.* | *2.–3.* | *3.–4.* | *1.* | *2.* | *3.* | *4.* | *1.* | *2.* | *3.* | *4.* |
| 1 | 28 | 27 | 29 | 100 | 31 | 0 | 0 | 43 | 19 | 10 | 31 |
| 2 | 28 | 28 | 28 | 100 | 5 | 0 | 0 | 18 | 25 | 19 | 15 |
| 3 | 28 | 28 | 28 | 100 | 72 | 42 | 22 | 33 | 37 | 34 | 30 |
| 4 | 28 | 28 | 35 | 100 | 28 | 12 | 30 | 18 | 6 | 13 | 4 |
| 5 | 28 | 28 | 28 | 100 | 40 | 52 | 25 | 26 | 12 | 15 | 12 |
| 6 | 28 | 28 | 28 | 100 | 89 | 55 | 59 | 44 | 30 | 43 | 30 |
| 7 | 28 | 28 | 28 | 100 | 50 | 60 | 43 | 35 | 6 | 13 | 6 |
| 8 | 28 | 28 | 28 | 100 | 42 | 24 | 92 | 41 | 37 | 30 | 22 |
| 9 | 21 | 35 | 28 | 100 | 26 | 35 | 43 | 29 | 29 | 12 | 11 |
| 10 | 28 | 28 | 28 | 100 | 86 | 98 | 63 | 40 | 33 | 23 | 13 |
| 11 | 28 | 28 | 28 | 100 | 0 | 17 | 17 | 20 | 8 | 10 | 8 |
| 12 | 28 | 56 | 77 | 100 | 107 | 135 | 362 | 35 | 37 | 47 | 34 |
| 13 | 28 | 28 | 28 | 100 | 87 | 123 | 156 | 37 | 43 | 43 | 43 |
| 14 | 28 | 28 | 28 | 100 | 97 | 146 | 259 | 35 | 46 | 43 | 38 |
| 15L | 28 | 28 | 28 | 100 | 187 | 245 | 212 | 41 | 43 | 37 | 33 |
| 15R |  |  |  | 100 | 135 | 86 | 112 |  |  |  |  |
| 16 | 28 | 28 | 28 | 100 | 177 | 255 | 74 | 32 | 30 | 35 | 33 |
| 17 | 29 | 28 | 28 | 100 | 234 | 176 | 207 | 24 | 27 | 23 | 23 |
| 18 | 35 | 28 | 28 | 100 | 191 | 163 | 96 | 14 | 12 | 17 | 19 |
| 19 | 28 | 28 | 28 | 100 | 107 | 116 | 71 | 46 | 40 | 41 | 42 |
| 20 | 28 | 34 | 22 | 100 | 96 | 83 | 28 | 34 | 34 | 35 | 30 |
| 21L | 28 | 28 | 49 | 100 | 182 | 210 | 226 | 28 | 37 | 29 | 35 |
| 21R |  |  |  | 100 | 90 | 92 | 94 |  |  |  |  |
| 22 | 28 | 28 | 28 | 100 | 64 | 45 | 51 | 18 | 26 | 13 | 20 |
