# Supplementary material for: Autologous Platelet- and Extracellular Vesicle-Rich Plasma Is an Effective Treatment Modality for Chronic Postoperative Temporal Bone Cavity Inflammation: Randomized Controlled Clinical Trial
Source: Front Bioeng Biotechnol. 2021 Jul 7;9:677541. doi: 10.3389/fbioe.2021.677541 (PMC8294456; doi:10.3389/fbioe.2021.677541)

Supplementary Material 10

# Chronic postoperative temporal bone cavity inflammation focus area measurement


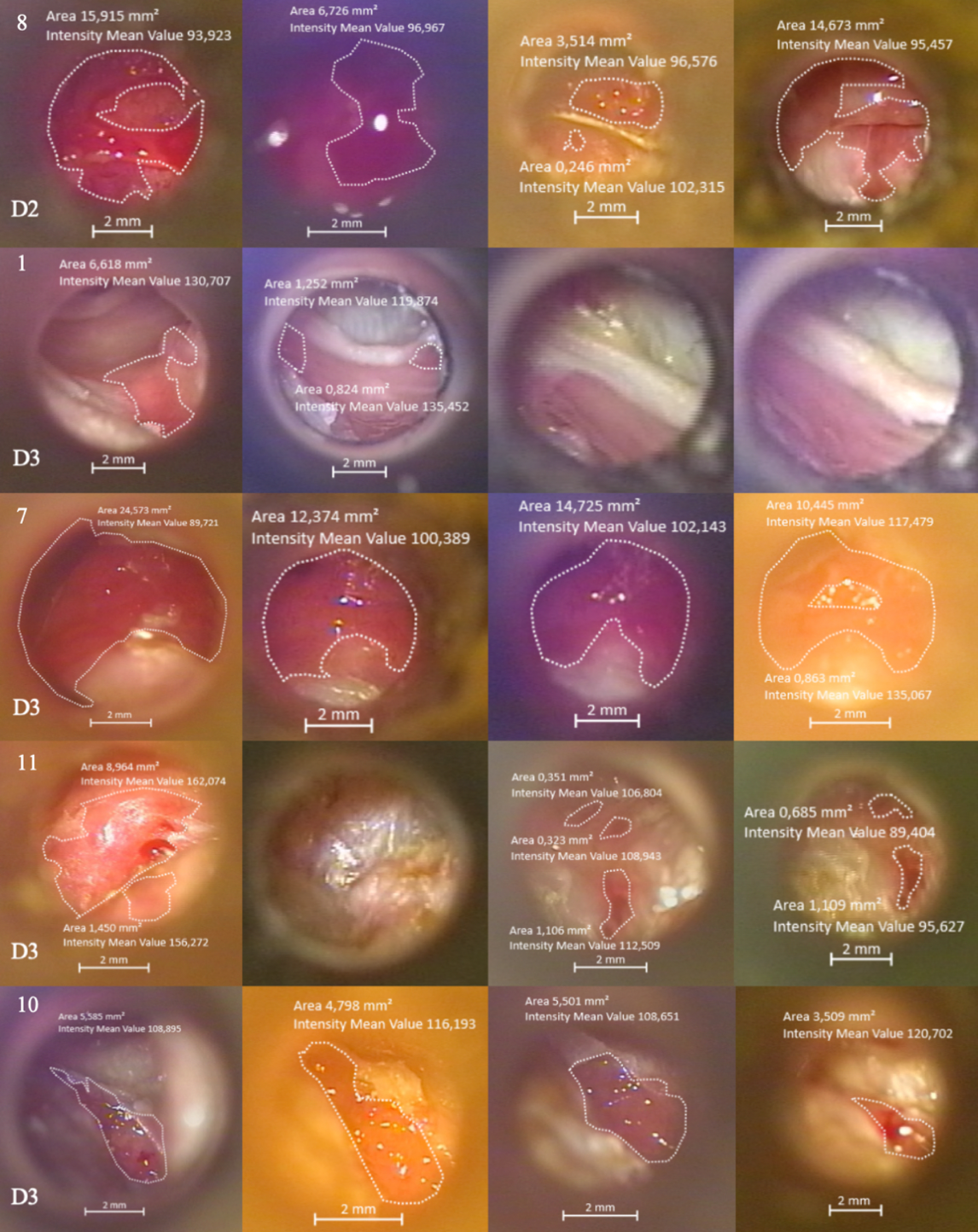


Figure: Chronic postoperative temporal bone cavity inflammation (CPTBCI) focus areas of patients treated with platelet- and extracellular vesicle-rich plasma (PVRP). Otomicroscopical photographs of one case of CPTBCI are shown in one row. Photographs captured from the 1. to the 4. check-up. are shown from left to right in each row. Areas (in mm^2^) and classifications of CPTBCI foci (in the lower-left corner of each row) are provided on each photograph. The patient's ID that matches the data in tables from Supplementary Material is shown in each row's top left corner.

Continues on the next page

Continuation of the previous Figure


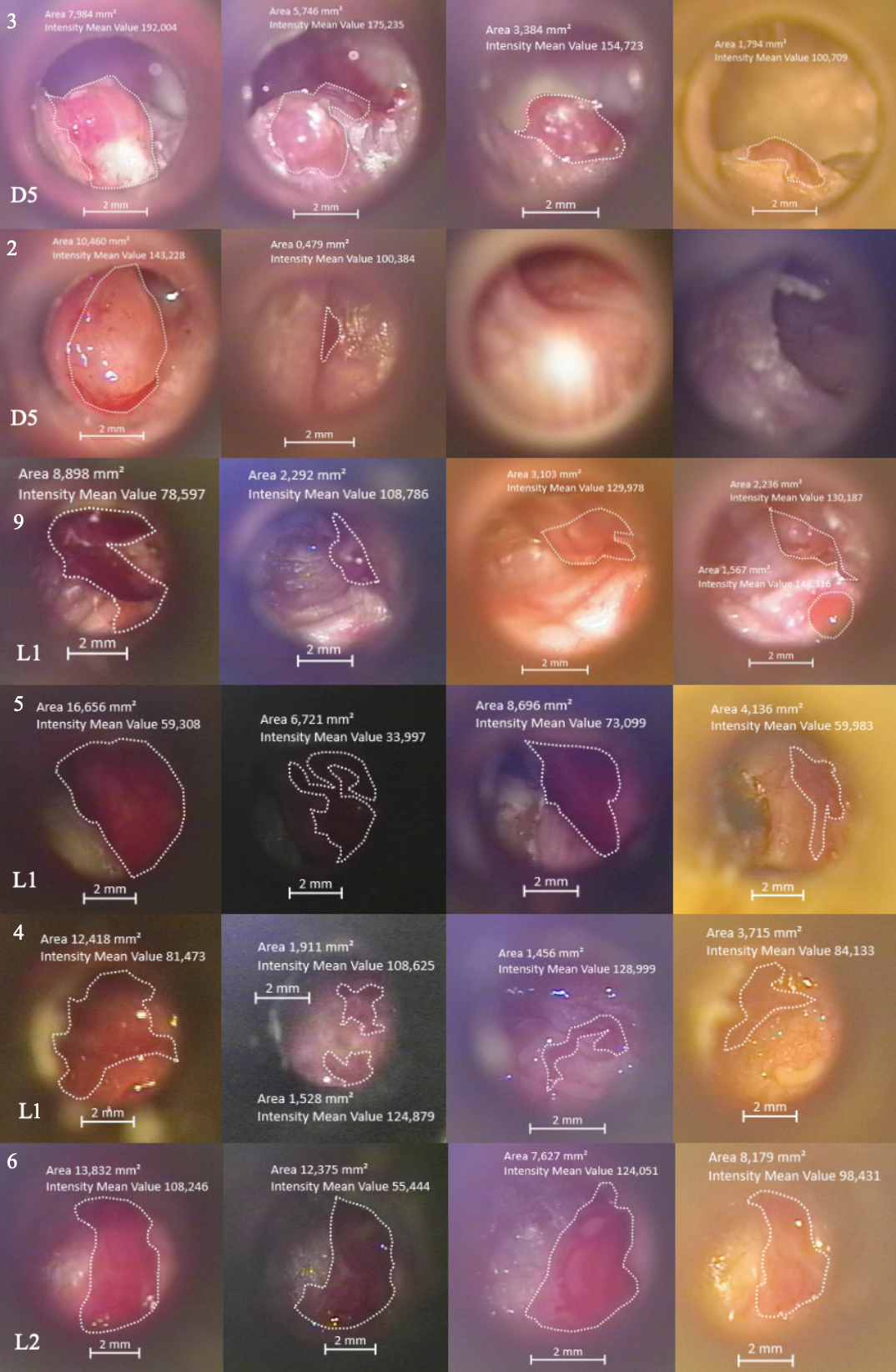


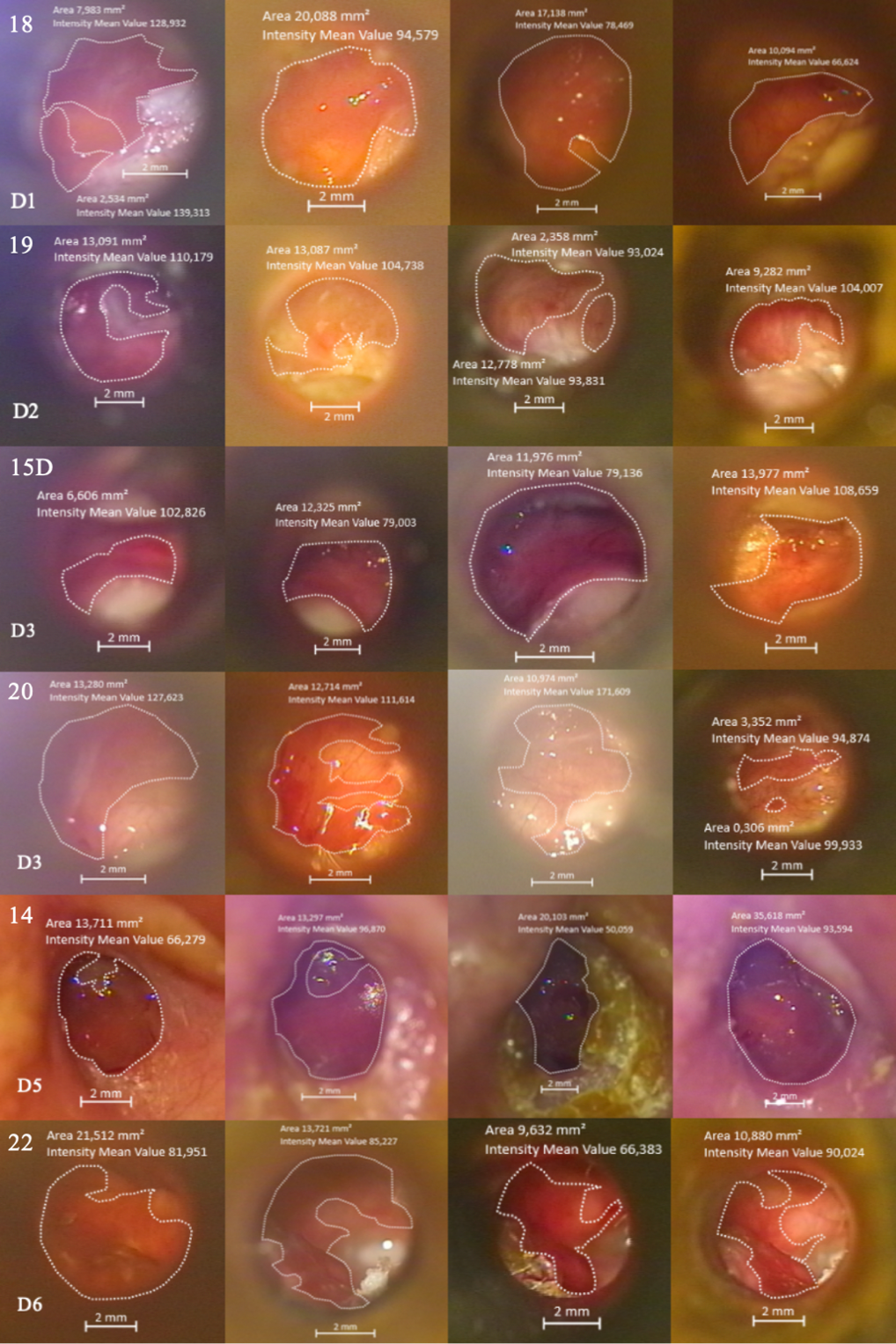


Figure: Chronic postoperative temporal bone cavity inflammation (CPTBCI) focus areas of patients treated with standard conservative measures. Otomicroscopical photographs of one case of CPTBCI are shown in one row. Photographs captured from the 1. to the 4. check-up. are shown from left to right in each row. Areas (in mm^2^) and classifications of CPTBCI foci (in the lower-left corner of each row) are provided on each photograph. The patient's ID that matches the data in tables from Supplementary Material is shown in each row's top left corner. Patients 15 and 21 had bilateral involvement.

Continues on the next page

Continuation of the previous Figure


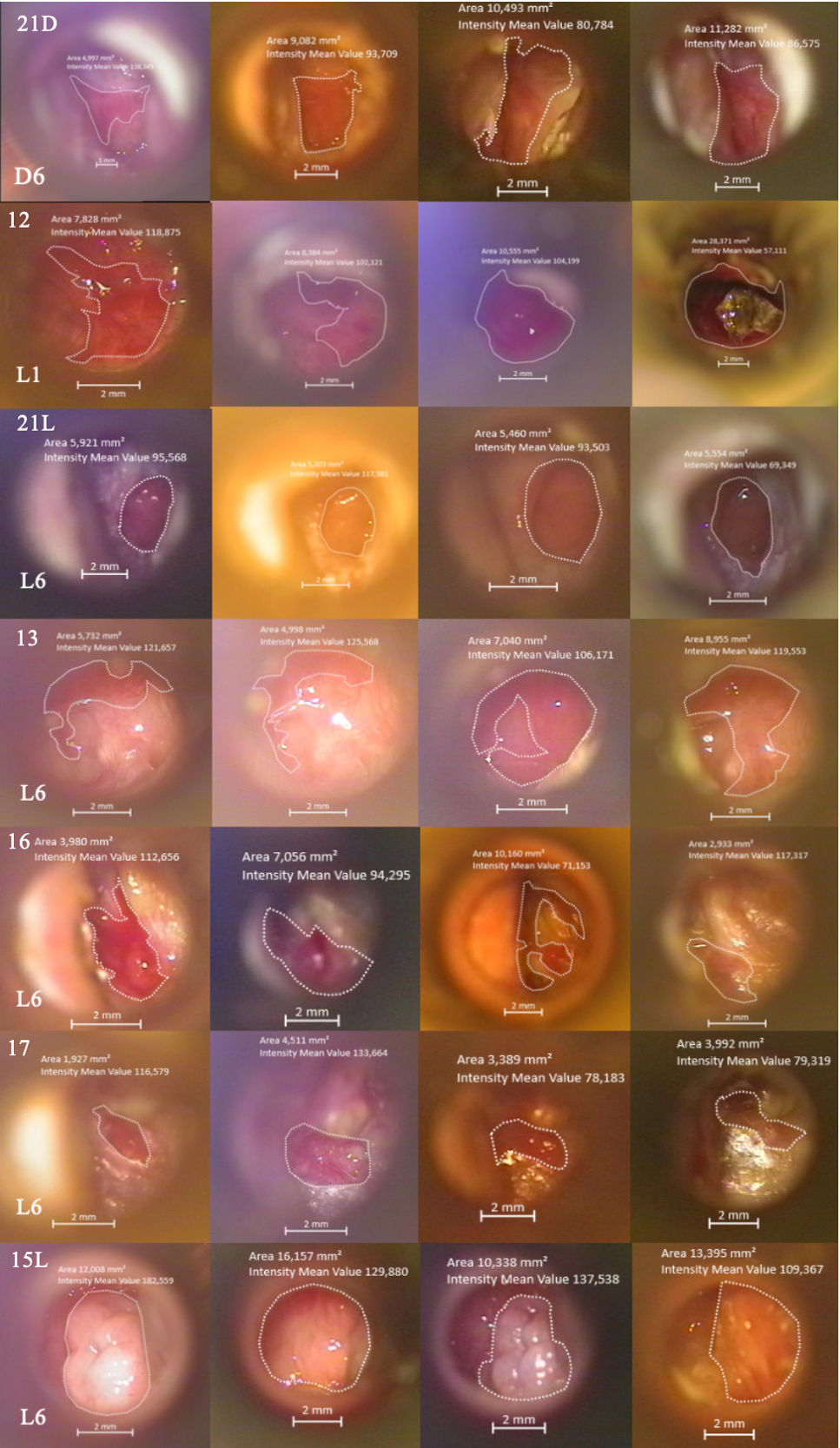

Supplement: Supplementary file 10 [file Table_10.DOCX]
